# Introducing pulse oximetry for outpatient management of childhood pneumonia: An implementation research adopting a district implementation model in selected rural facilities in Bangladesh

**DOI:** 10.1016/j.eclinm.2022.101511

**Published:** 2022-06-29

**Authors:** Ahmed Ehsanur Rahman, Shafiqul Ameen, Aniqa Tasnim Hossain, Janet Perkins, Sabrina Jabeen, Tamanna Majid, AFM Azim Uddin, Md. Ziaul Haque Shaikh, Muhammad Shariful Islam, Md. Jahurul Islam, Sabina Ashrafee, Husam Md. Shah Alam, Ashfia Saberin, Sabbir Ahmed, Goutom Banik, ANM Ehtesham Kabir, Anisuddin Ahmed, Mohammod Jobayer Chisti, Steve Cunningham, David H Dockrell, Harish Nair, Shams El Arifeen, Harry Campbell

**Affiliations:** a NIHR Global Health Research Unit on Respiratory Health (RESPIRE), Usher Institute, The University of Edinburgh, Edinburgh, UK; b International Centre for Diarrhoeal Disease Research, Bangladesh, (icddr,b), Dhaka, Bangladesh; c Directorate General of Health Services, Ministry of Health and Family Welfare, Government of Bangladesh, Bangladesh; d Save The Children, Dhaka, Bangladesh

**Keywords:** Pulse oximetry, IMCI, Pneumonia, Feasibility, Implementation research, Bangladesh

## Abstract

**Background:**

Pulse oximetry has potential for identifying hypoxaemic pneumonia and substantially reducing under-five deaths in low- and middle-income countries (LMICs) setting. However, there are few examples of introducing pulse oximetry in resource-constrained paediatric outpatient settings, such as Integrated Management of Childhood Illness (IMCI) services.

**Methods:**

The National IMCI-programme of Bangladesh designed and developed a district implementation model for introducing pulse oximetry in routine IMCI services through stakeholder engagement and demonstrated the model in Kushtia district adopting a health system strengthening approach. Between December 2020 and June 2021, two rounds of assessment were conducted based on WHO's implementation research framework and outcome variables, involving 22 IMCI service-providers and 1680 children presenting with cough/difficulty-in-breathing in 12 health facilities. The data collection procedures included structured-observations, re-assessments, interviews, and data-extraction by trained study personnel.

**Findings:**

We observed that IMCI service-providers conducted pulse oximetry assessments on all eligible children in routine outpatient settings, of which 99% of assessments were successful; 85% (95% CI 83,87) in one attempt, and 69% (95% CI 67,71) within one minute. The adherence to standard operating procedure related to pulse oximetry was 92% (95% CI 91,93), and agreement regarding identifying hypoxaemia was 97% (95% CI 96,98). The median performance-time was 36 seconds (IQR 20,75), which was longer among younger children (2-11 months: 44s, IQR 22,78; 12-59 months: 30s, IQR 18,53, *p* < 0.01) and among those classified as pneumonia/severe-pneumonia than as no-pneumonia (41s, IQR 22,70; 32s, IQR 20,62, *p* < 0.01). We observed improvements in almost all indicators in round-2. IMCI service-providers and caregivers showed positive attitudes towards using this novel technology for assessing their children.

**Interpretation:**

This implementation research study suggested the adoption, feasibility, fidelity, appropriateness, acceptability, and sustainability of pulse oximetry introduction in routine IMCI services in resource-poor settings. The learning may inform the evidence-based scale-up of pulse oximetry linked with an oxygen delivery system in Bangladesh and other LMICs.

**Funding:**

This research was funded by the UK National Institute for Health Research (NIHR) (Global Health Research Unit on Respiratory Health (RESPIRE); 16/136/109) using UK aid from the UK Government to support global health research.

**Research in context**

**Evidence before this study**

We conducted a literature review to summarise the experiences of introducing and integrating pulse oximetry in resource-constrained paediatric outpatient settings. We searched in Medline, PubMed and Web of Science databases with the following search strategy: "pulse oximetry" AND "implementation outcome variables such as appropriateness, acceptability, adoption, adherence, coverage, feasibility, fidelity, performance, sustainability, usability, utility, impact" AND "low-and middle-income countries". All studies published from 01 January 2014, to 03 February, 2022 were included. Several studies have reported various aspects of introducing and performing pulse oximetry, but few adopted a comprehensive approach assessing the implementation aspects in real-life settings based on WHO-recommended implementation outcome variables. We have identified several studies on performance of pulse oximetry assessment for childhood pneumonia, but these studies were mostly implementation research conducted in clinical settings.

**Added value of this study**

We adopted a health system strengthening approach for introducing pulse oximetry in routine Integrated Management of Childhood Illness (IMCI) services in Bangladesh and developed a district model of implementation under government leadership with active involvement and engagement of national- and district-level stakeholders and service-providers. We conducted the implementation research based on WHO's implementation research framework, where the research questions were finalised in consultation with the National IMCI-programme, district managers and IMCI service-providers. This is to the best of our knowledge the first study reporting most of WHO's implementation outcome variables related to pulse oximetry introduction in routine outpatient settings by government-employed IMCI service-providers.

**Implications of all the available evidence**

Our study suggested that the district implementation model through a health system strengthening approach was successful as the IMCI service-providers adopted the new technology and could perform pulse oximetry successfully, efficiently and accurately without facing significant difficulties. The policymakers can consider from this experience to take an evidence-based decision for integration and scale-up of pulse oximetry in routine IMCI services with an improved oxygen delivery system in Bangladesh and other low- middle-income countries.

Alt-text: Unlabelled box

## Introduction

Pneumonia is the leading cause of childhood mortality, accounting for approximately 16% of under-five deaths globally.^1^ The majority of these deaths occur in low- and middle-income countries (LMICs) and most are preventable.^1^, ^2^, ^3^ It takes more lives than any other causes in post-neonatal periods, specially in low-resource settings.^4^ In Bangladesh, it causes approximately one million episodes of severe-illness and 24,000 under-five deaths every year.^5^^,^^6^ Around 30% children with pneumonia suffer from hypoxaemia, defined by low oxygen saturation in arterial blood (SpO_2_), and is a strong predictor of hospitalisations and deaths.^7^^,^^8^ Pulse oximetry is an accurate and non-invasive procedure to measure SpO_2_ at point of care.^9^ It can significantly improve childhood pneumonia classification through hypoxaemia identification and substantially reduce deaths in LMIC settings.^9^

In 2014, the World Health Organization (WHO) updated its outpatient-based child-curative care guidelines, i.e., Integrated Management of Childhood Illness (IMCI) and recommended introducing pulse oximetry for pneumonia assessment.^10^ Several health system challenges are associated with integrating a new technology/device like pulse oximetry in LMIC settings.^11^ Although there is some published experience of introducing pulse oximetry in low-resource settings, most predates the 2014-WHO recommendations.^12^, ^13^, ^14^

Successful introduction of a generic recommendation, technology or device in routine services demands context-specific adaptations, demonstration and feasibility assessments.^15^, ^16^, ^17^ In 2019, the national IMCI-programme of the Government of Bangladesh (GoB) decided to introduce pulse oximetry in routine IMCI services.^18^ A national technical committee was formed, which updated the National IMCI Implementation Package by incorporating recommendations to conduct pulse oximetry assessments on all children presenting with cough/difficulty-in-breathing for pneumonia assessments.^18^^,^^19^ The IMCI-programme also decided to demonstrate the updated package in a district setting to inform evidence-based scale-up. Here, we present the experience and learning synthesised during the demonstration phase.

## Methods

### Study design

An implementation research study was conducted, where the IMCI-programme designed, developed, and demonstrated a district implementation model to introduce pulse oximetry in routine IMCI services. icddr,b, an international health research organisation based in Bangladesh, provided implementation facilitation support and assessments.

### Study settings

This study was conducted in Kushtia district (zila), approximately 200 kilometres from Dhaka, which was selected as the demonstration site as its under-five mortality rate was close to the national average and the IMCI services were functioning relatively well.^17^ The IMCI-programme, in consultation with the district manager (Civil Surgeon) and sub-district managers (Upazila Health and Family Planning Officers), selected one district hospital (secondary-referral), all five sub-district hospitals (primary-referral) and six union-based facilities (health centres) as demonstration facilities (additional details in SM1&SM2).

### District implementation model

The IMCI-programme designed and developed a district implementation model through stakeholder engagement and adopted a health systems strengthening approach for improving the overall IMCI services, including the introduction of pulse oximetry in demonstration facilities (additional details in SM3). It had four components, which are as follows:

i) Sensitisation of district- and sub-district managers and IMCI service-providers through workshops and involve them in planning and implementation
ii) Capacity development of IMCI service-providers, supervisors, data officers and district- and sub-district managers
iii) Health system strengthening to improve IMCI service readiness and distribution of pulse oximetry devices through the routine distribution channel
iv) Follow-up support to IMCI service-providers through routine monitoring, supportive supervision and performance appraisal workshops

The demonstration study started in October-2019, was suspended in March-2020 due to COVID-19, resumed in October-2020, and ended in October-2021.

i. **Sensitisation and Planning**

The National IMCI-programme organised a district-level workshop with the district- and sub-district managers of Kushtia to sensitise them regarding the importance of introducing pulse oximetry in routine IMCI services and develop a high-level plan for implementing the updated IMCI implementation package in the selected health facilities. Following that, a workshop was organised in each sub-district with the respective sub-district health managers, IMCI services-providers and their clinical supervisors for further sensitisation and developing facility-specific micro-plans. Then, a workshop was also organised in each of the selected health facilities with IMCI services-providers and their supervisors to introduce pulse oximetry officially. These workshops were organised between October and December 2019. Unfortunately, the implementation was suspended in March 2020 due to the COVID-19 pandemic. The IMCI-programme decided to resume the activities in September 2020. Between October and November 2020, another round of district- and sub-district-level workshops were organised to update the high-level as well as the facility-specific micro plans.

ii. **Capacity building**

Between December 2019 and February 2020, the IMCI Programme organised a series of training for IMCI service-providers from across the country based on the updated IMCI implementation package as a part of their routine programme activities. The National IMCI master trainers facilitated the sessions, and all IMCI service-providers from the selected health facilities of Kushtia received the training. The updated IMCI training manual had a module on pneumonia assessment and pulse oximetry adapted from the pulse oximetry training manual of WHO. The module had information and instructions on the pathophysiology of hypoxaemia; function, types and parts of a pulse oximetry device; steps of conducting pulse oximetry assessments; and maintenance of the device. Half a day was dedicated to pulse oximetry which included both theoretical ad practical sessions. In addition, an orientation session was organised in Kushtia for the district- and sub-district managers, IMCI clinical supervisors, and data officers to discuss the basics of pulse oximetry and inclusion of SpO_2_ status and hypoxaemia reporting in the IMCI service register and monthly reporting form.

iii. **Distribution of pulse oximeters**

icddr,b surveyed the selected health facilities in December 2019 and identified the gaps in the availability of equipment, drugs, and logistics required for managing pneumonia based on the updated IMCI guidelines. The IMCI-programme addressed these gaps through the existing procurement and distribution channel. In addition, icddr,b supported the IMCI-programme to procure and distribute Masimo Rad-5v handheld pulse oximeters to the selected health facilities. The specific model was chosen by the national technical committee (mentioned earlier) based on the superiority in technical specifications and expert consultations. The distribution of pulse oximeters and other logistics were done between January and February 2020. In addition, batteries of the pulse oximetry devices were replaced through the existing procurement and distribution channel throughout the implementation period. In general, batteries were replaced after 15-25 days in the district hospitals, 30-40 days in sub-district hospitals and 60-70 days in health centres.

iv. **Monitoring, supervision and performance review workshops**

National, district and sub-district managers were responsible for monitoring and supervising the pulse oximetry related implementation in Kushtia. Representatives from the IMCI-programme conducted several field visits during the implementation period. They also conducted online meetings with the district- and sub-district managers to review the progress and discuss implementation quality and challenges. In addition, the district and sub-district managers conducted at least one supervisory visit to each selected facility during the implementation period. The observations from these supervisory visits were discussed in the routine monthly meetings. Moreover, a review workshop was organised in March 2021 with all IMCI service-providers in the presence of national-, district- and sub-district managers and clinical supervisors to discuss the achievements and gaps identified through the first round of assessments. A similar review workshop was also organised in October 2021 after completing the second round of assessments.

### Study objectives

We adapted WHO's implementation research framework and implementation outcome variables for this study.^20^ The IMCI-programme and icddr,b jointly conducted a series of workshops with district- and sub-district managers to finalise the research questions based on WHO's framework and set a benchmark for successful demonstration for each outcome variable (additional details in SM4).^18^ The primary research questions are outlined in Table 1.

**Table 1:** Research questions and benchmarks for successful demonstration; adapted from WHO's implementation outcome variables and finalised through stakeholder consultations.

WHO's framework	Research questions	Proposed indicator	Benchmark
a) Adoption	I. Use: Do IMCI service-providers conduct pulse oximetry assessments?	Proportion of children presenting with cough/difficulty-in-breathing assessed by IMCI service-providers with a pulse oximetry device	>90%
b) Feasibility	II. Success: Can IMCI service-providers successfully conduct pulse oximetry assessments?	Proportion of children presenting with cough/difficulty-in-breathing assessed by IMCI service-providers with a pulse oximetry device and obtain a stable SpO_2_ reading	>80%
	III. Usability by attempt: Can IMCI service-providers successfully conduct pulse oximetry assessments in the first attempt?	Proportion of children presenting with cough/difficulty-in-breathing assessed by IMCI service-providers with a pulse oximetry device and obtain a stable SpO_2_ reading in one attempt	>75%
	IV. Usability by time: Can IMCI service-providers successfully perform pulse oximetry in one minute?	Proportion of children presenting with cough/difficulty-in-breathing assessed by IMCI service-providers with a pulse oximetry device and obtain a stable SpO_2_ reading in one minute	>66%
c) Fidelity	V. Adherence: Do IMCI service-providers follow Standard Operating Procedure (SoP) while conducting pulse oximetry assessments?	Proportion of children presenting with cough/difficulty-in-breathing assessed by IMCI service-provider with a pulse oximetry device by putting the probe in appropriate position and direction and taking measures to keep the child calm during the procedure	>75%
	VI. Agreement: Can IMCI service-providers identify hypoxaemia through pulse oximetry?	Level of observed agreement between IMCI service-providers and study nurses regarding hypoxaemia identification through pulse oximetry	>90%
d) Appropriateness	VII. Experience: Do IMCI service- providers conduct pulse oximetry assessments with reasonably low barriers and challenges?	Proportion of IMCI service-providers reporting an average challenge level of 80% or less regarding conducting pulse oximetry assessments in routine outpatient settings	>80%
e) Acceptability	VIII. Usefulness: Do IMCI service-providers perceive pulse oximetry as useful?	Proportion of IMCI service-providers reporting that pulse oximetry is useful for identifying hypoxaemia and pneumonia classification	>80%
	IX. Importance: Do the caregivers perceive pulse oximetry as important?	Proportion of caregiver of children presenting with cough/difficulty-in-breathing assessed by IMCI service-providers with a pulse oximetry device reporting that pulse oximetry was important for assessing their children	>80%
	X. Satisfaction: Are the caregivers satisfied with pulse oximetry introduction in routine IMCI services?	Proportion of children presenting with cough/difficulty-in-breathing assessed by IMCI service-providers with a pulse oximetry device reporting that they will allow conducting pulse oximetry assessments on their children in future visits.	>80%
f) Sustainability	XI. Sustainability: Does the pulse oximetry performance of IMCI service-providers sustain over time (rounds)?	Proportions of the above-mentioned indicators representing adoption, feasibility, fidelity, appropriateness and acceptance of pulse oximetry demonstrating equal or better performance round-2 than that of round-1	>80%

### Study participants

Children aged 2-59 months presenting with cough/difficulty-in-breathing and receiving IMCI services at selected health facilities and government-employed IMCI service-providers (doctors, nurses, and paramedics) providing those services were the study population.

### Sampling

We calculated the sample size for each of the primary research questions based on the benchmark set for successful demonstration (Table 1). The maximum variance (67%) was expected for the usability-time indicator. The adjusted sample size was 639 children presenting with cough/difficulty-in-breathing per round. There were 22 IMCI service-providers in the selected facilities. We aimed to assess the pulse oximetry performance of all IMCI service-providers by observing and re-assessing at least 30 children presenting with cough/difficulty-in-breathing per provider, per round. We approached all children presenting with cough/difficulty-in-breathing on the day of assessment, and it required 3-7 days per provider per round to achieve the required sample size. On the day the required sample size (30 children) was reached for each IMCI service-provider, we continued enrolment and data collection until the end of business hours.

### Data collection

We conducted two rounds of assessment: round-1 in December 2020-February 2021; round-2 in March-May 2021. The data collection procedures included structured-observation of IMCI service-providers conducting pulse oximetry assessments during routine consultations by study nurses, re-assessment (history taking, clinical assessment and pulse oximetry) of children (after IMCI consultation) by study nurses, exit-interviews of the caregivers by study paramedics, data-extraction from IMCI-registers by data collectors, and structured-interviews with IMCI service-providers by study paramedics (additional details in SM5). All respondents gave written informed consent prior to participating. We assessed the pulse oximetry performance of the same IMCI service-providers in both rounds. All forms/questionnaires were pretested separately on at least five participants. The construct/framing of some of the questions and skip logics were updated based on the pretests. Data collection was conducted by means of a specially designed android-app, which had the provision to capture each pulse oximetry step with time stamping.^21^ The data collection team received extensive training and refresher training on IMCI guidelines, pulse oximetry principles and process, data collection forms/questionnaires and the data-collection app. All tools were developed based on the extensive literature review and the structured-interview and exit-interview tool for the IMCI service-providers on barriers and operational challenges were developed based on qualitative exploration. All tools were field-tested before finalisation.

i. **Enrolment**

A non-medical study staff approached all children in the waiting room/area of selected health facilities and enrolled children aged 2-59 presenting with cough/difficulty-in-breathing before receiving consultation from the IMCI service-providers.

ii. **Structured-observation**

Trained study nurses conducted structured-observations of IMCI service-providers conducting pulse oximetry assessments on children presenting with cough/ difficulty-in-breathing. The observers collected information on the following outcomes: use, success, usability by attempts, usability by time and performance time. The observers received ten days of training before data collection (five days on IMCI, one day on pulse oximetry, two days on using the data collection app based on the structured-observation tool, and two days of field practice). Three days of refresher training was organised before the second round of data collection.

iii. **Re-assessment**

A separate group of trained study nurses conducted re-assessments of the enrolled children in a separate room after the structured-observation. The assessors collected data on the history of illness, conducted clinical assessments based on the IMCI guideline, and conducted pulse oximetry assessments using a device of the same model. The assessors received ten days of training before data collection (five days on IMCI, one day on pulse oximetry, two days on the re-assessment tool, and two days for field practice). Three days of refresher training was organised before the second round of data collection.

iv. **Exit-interview**

Trained paramedics conducted exit-interviews of the caregivers of the enrolled children after re-assessment. The interviewers used an interviewer-administered structured-questionnaire (translated in Bangla) to collect data on the acceptability and perceived importance of pulse oximetry. The interviewers received five days of training before data collection (one day on pulse oximetry, two days on the exit-interview questionnaire and two days of field practice). One day of refresher training was organised before the second round of data collection.

v. **Data-extraction**

Trained data collectors extracted data from the IMCI services register of those children who were enrolled in this study. The data collectors took snapshots of the IMCI registers after service hours on the day of assessment (additional details in SM6). We obtained administrative approval from national IMCI-programme of Directorate General of Health Services of Bangladesh for conducting this study including the approval for taking snapshots of register. We also received approval from district-level health manager at Kushtia district. Furthermore, we have conducted facility level sensitisation, preparatory and planning meeting in presence of the IMCI service-providers, respective supervisors and facility managers. A dedicated team of data operators used a desktop-based application that was specially developed for this study and entered all available information, including SpO_2_ and hypoxaemia status reported in the IMCI registers. The data operators received two days of training on IMCI registers and the desktop-based application. A data entry supervisor checked the data-extraction process and conducted re-extraction and re-entry of 5% observations. There were very few cases where we identified minor issues, which we rectified by checking the source documents (snapshopts).

vi. **Structured-interviews**

The interviewers who conducted the exit-interviews of the caregivers also conducted structured-interviews with IMCI services-providers at the end of round-2. The interviewers used a structured-questionnaire with a five-point Likert scale and collected data on the perceived utility of introducing pulse oximetry in routine IMCI services and barriers and challenges faced while conducting pulse oximetry in routine practice.

vii. **Monitoring and supervision**

A medical graduate directly supervised the data collection team. During the data collection period, the supervisor conducted regular monitoring visits meetings and organised weekly meetings with the data collection team to discuss data quality issues and specific gaps.

### Analysis plan

The following indicators were selected as primary outcomes of interest: adoption-use, feasibility-success, feasibility-usability by attempt, feasibility-usability by time, fidelity-SoP adherence, fidelity-agreement, appropriateness-experience, acceptability-usefulness, acceptability-importance, and acceptability-satisfaction (Table 1).

Adoption was operationally defined as conducting pulse oximetry assessments on children presenting with cough/difficulty-in-breathing. A pulse oximetry assessment was considered successful if a stable (±1%) SpO_2_ reading was observed for at least 10 seconds with adequate signal strength. Performance time was defined by the time taken (in seconds) from placing the probe to obtaining a stable reading. Adherence to standard operating procedures (SoP-adherence) was defined as putting the probe in the appropriate position and direction, and taking measures to keep the baby calm before conducting pulse oximetry assessments. The agreement was reported on identifying hypoxaemia through pulse oximetry by IMCI service-providers and re-assessment study nurses. We used a SpO_2_ cut-off of <94% to operationally define hypoxaemia.^22^ Regarding experience, IMCI service-providers reported barriers and challenges on a five-point Likert scale for 12 device-, patient-, environment-, and user-related factors.

Relationships between patient- (age, sex, weight-for-height, clinical features, and pneumonia status), provider- (age, sex, and designation), facility-related (types) factors and assessment rounds, and primary outcomes of interest (Table 1) were considered as the secondary outcomes of interest (additional details in SM4). We also reported median performance time and infection prevention and control (IPC) practice, defined as cleaning the probe or attachment site of the child before conducting pulse oximetry assessments, as secondary outcomes of interest.

For categorical variables as outcomes of interest, we presented point estimates with 95% confidence intervals (CI). We used prevalence-adjusted and bias-adjusted kappa (PABAK) to report the agreement between IMCI service-providers and re-assessment study nurses.^23^ We used Generalised Estimating Equation (GEE) models and reported crude and adjusted odds ratios (with 95% CI and p-value) to assess the adjusted effect of various factors and assessment rounds on these indicators. Wald statistics were used for checking the adequacy of the models. We used radar-plots to present the variations by each IMCI service-provider and reported the heterogeneity with I^2^ and Tau^2^ statistics. The statistical significance of the estimates have been reported at 5% level of significance.

For performance time, we checked the normality distribution using Shapiro-Wilk test (SM7). As they were non-normally distributed, we presented medians with interquartile ranges (IQRs) and used Mood's non-parametric equality of medians test to check for significant differences in various factors and assessment rounds. Barrier and challenge experiences were presented as means with standard deviations (SDs). We used Stata version 15.0 for data analysis. More details regarding the analysis plan are available in the supplementary material (SM4).

Ethical approval of the study was obtained from the Institutional Review Board of icddr,b (Protocol Number: PR-18054). Research Governance of The University of Edinburgh declared the study does not need any UK based ethical opinion as it was categorised as minimal risk. All respondents gave written informed consent prior to participating.

## Role of the funding source

The funders had no role in study design, data collection, data analysis, data interpretation, or writing the report. AER, SA and ATH had access to all data and take full responsibility for its integrity and the accuracy of the analyses. The corresponding author had final responsibility for the decision to submit for publication.

## Results

### Sample size by each round of assessments

**Figure 1:**
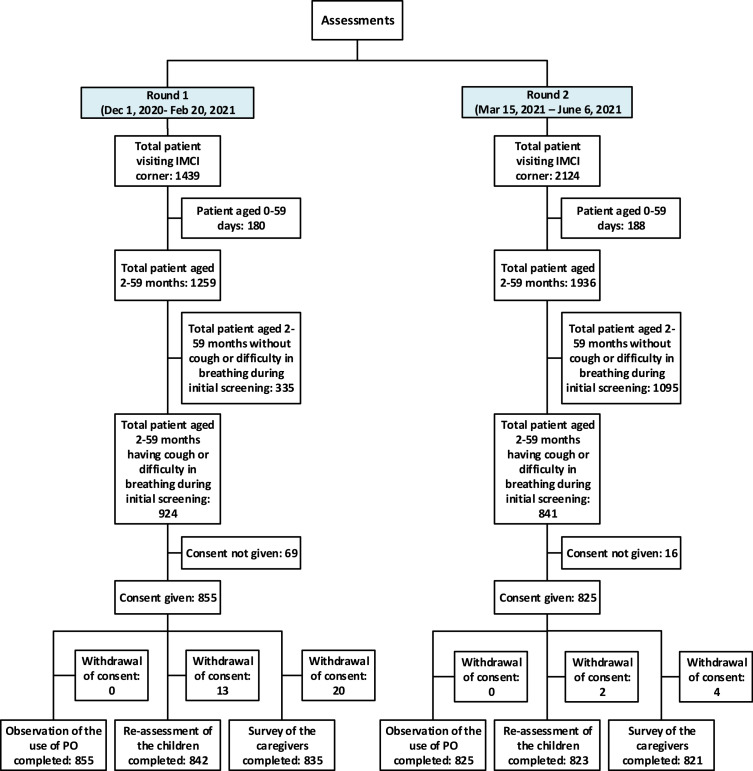
Sample size by each round of assessments.

We assessed the pulse oximetry performance of five doctors, five nurses and twelve paramedics as IMCI service-providers in both rounds (additional details in SM8). During the two rounds of assessment, 3563 children received IMCI services from the selected facilities, of which 3195 (90%) were aged 2-59 months, 1765 (49%) had cough/difficulty-in-breathing, and 1680 (47%) were enrolled for assessments (Figure 1). Around one percent (0.9%) enrolled children were not re-assessed and 1.4% caregivers could not be interviewed.

Of the enrolled children, 729 (43%) were aged 2-11 months, 164 (10%) were wasted/severely-wasted, and 220 (13%) were classified as pneumonia/severe-pneumonia (additional details in SM9, SM10, &SM11).

### Performance of conducting pulse oximetry assessments by IMCI service-providers

**Figure 2:**
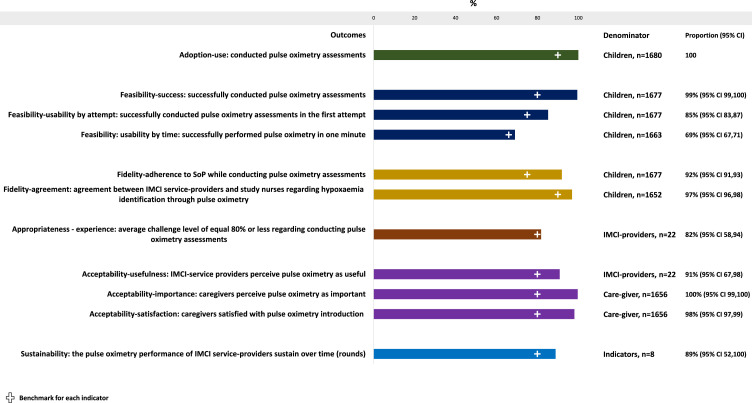
Performance of conducting pulse oximetry assessments by IMCI service-providers among children presenting with cough/difficulty-in-breathing, presented in percentages with 95% CI, (N=1680). Each of the bars indicate a distinct implementation outcome indicator. The first green bar represents adoption, the blue bars represent feasibility, the yellow bars represent fidelity, the brown bar represents appropriateness, the purple bars represent acceptability and the light blue bar at the end represents sustainability.

During our assessment, the IMCI service-providers conducted pulse oximetry assessments (adoption-use) on all children presenting with cough/difficulty-in-breathing, of which 99% of assessments were successful (feasibility-success); 85% (95% CI 83,87) in one attempt (feasibility-usability by attempt) and 69% (95% CI 67,71) within one minute (feasibility-usability by time) (Figure 2). The adherence to SoPs was 92% (95% CI 91,93) (fidelity-SoP-adherence), and the agreement was 97% (95% CI 96,98) (fidelity-agreement). All IMCI service-providers reported that pulse oximetry was useful for identifying hypoxaemia and pneumonia classification (acceptability-usefulness). Almost all (98%) caregivers felt that it was an important diagnostic for assessing their children (acceptability-importance) and all of them would allow conducting it on their children during future visits (acceptability-satisfaction). The indicators representing adoption, feasibility, fidelity, appropriateness, and acceptance demonstrated equal or better performance in round-2 (sustainability).

### Influence of patient-,provider- and facility-related factors on the performance of conducting pulse oximetry assessments

Table 2 summarises the effect of various factors and assessment rounds on feasibility- and fidelity-related indicators. The cells with asterisks indicate the statistically significant odds ratio. The odds of successfully conducting pulse oximetry assessments in one attempt was significantly higher among older (aged 12-59 months) (AOR 2.19, 95% CI 1.65, 2.92) and male children (AOR 1.54, 95% CI 1.16, 2.04) (Wald statistics 44.71, *p* < 0.01). They were also more likely to successfully conduct pulse oximetry assessments within one minute on older children (AOR 2.05, 95% CI 1.65, 2.55) (Wald statistics 59.98, *p* < 0.01). Older, female, and sub-district hospital- and health centre-based providers were less likely to adhere to pulse oximetry SoPs (Wald statistics 100.14, *p* < 0.01). Substantial improvement was observed in round-2 regarding most of these indicators. More details about the models are available in SM12, SM13, SM14 & SM15.

**Table 2:** Influence of patient-, provider- and facility-related factors on the performance of conducting pulse oximetry assessments by IMCI service-providers, presented in adjusted odds ratios, N=1680.

	Feasibility-usability: Success at first attempt	Feasibility-performance time: Success within 60 seconds	Fidelity-adherence: Adherence to SoP of PO use	Fidelity-agreement: Observed agreement
	AOR (95% CI)	AOR (95% CI)	AOR (95% CI)	AOR (95% CI)
Patient-related factors				
Age				
2-11 months	Ref	Ref	Ref	Ref
12-59 months	2.2 (1.65,2.93)*	2.05 (1.65,2.55)*	1.06 (0.69,1.61)	1.19 (0.67,2.11)
Sex				
Female	Ref	Ref	Ref	Ref
Male	1.54 (1.16,2.04)*	1.21 (0.98,1.5)	0.69 (0.46,1.05)	1.07 (0.6,1.89)
Z-score (weight for height)				
Not wasted (-2SD and above)	Ref	Ref	Ref	Ref
Wasted (below -2SD)	0.82 (0.47,1.43)	0.79 (0.51,1.24)	0.75 (0.29,1.94)	1.46 (0.36,5.95)
Severely wasted (below -3SD)	0.8 (0.41,1.55)	0.89 (0.52,1.53)	1.59 (0.33,7.72)	0.75 (0.21,2.59)
Fever				
No	Ref	Ref	Ref	Ref
Yes	0.99 (0.52,1.91)	0.79 (0.49,1.27)	0.5 (0.18,1.36)	0.92 (0.3,2.84)
Fast breathing				
No	Ref	Ref	Ref	Ref
Yes	1.03 (0.3,3.56)	1.08 (0.4,2.91)	0.6 (0.07,5.43)	1 (0.16,6.15)
Chest indrawing				
No	Ref	Ref	Ref	Ref
Yes	0.68 (0.28,1.65)	0.9 (0.44,1.84)	0.81 (0.19,3.55)	1.35 (0.33,5.53)
IMCI classification				
No pneumonia	Ref	Ref	Ref	Ref
Pneumonia/Severe pneumonia	1.06 (0.3,3.78)	0.88 (0.32,2.42)	1.32 (0.14,12.1)	0.38 (0.06,2.4)
Provider-related factors				
Age				
≤ 35 years	Ref	Ref	Ref	Ref
> 35 years	1.08 (0.72,1.62)	1.17 (0.9,1.53)	0.29 (0.15,0.53)*	1.1 (0.27,4.44)
Sex				
Female	Ref	Ref	Ref	Ref
Male	0.88 (0.54,1.44)	1.05 (0.78,1.43)	2.98 (1.34,6.63)*	0.72 (0.1,5.31)
Designation				
Doctors	Ref	Ref	Ref	Ref
Nurse	0.6 (0.34,1.05)	1.03 (0.73,1.45)	0.42 (0.16,1.1)*	0.49 (0.06,4.17)
Paramedics	0.82 (0.46,1.45)	0.77 (0.55,1.08)	1.88 (0.55,6.43)*	0.3 (0.04,2.12)
Facility-related factors				
District Hospital	Ref	Ref	Ref	Ref
Sub-District Hospital	0.91 (0.49,1.72)	1.06 (0.72,1.55)	0.03 (0,0.25)	1.33 (0.15,11.38)
Health Centre	0.76 (0.36,1.62)	0.81 (0.51,1.29)	0 (0,0.05)	4.97 (0.36,68.53)
Assessments				
Round				
Round 1	Ref	Ref	Ref	Ref
Round 2	0.98 (0.74,1.3)	1.3 (1.05,1.62)*	28.62 (11.03,74.29)*	1.76 (0.96,3.23)

### Variability in performance of conducting pulse oximetry assessments

**Figure 3:**
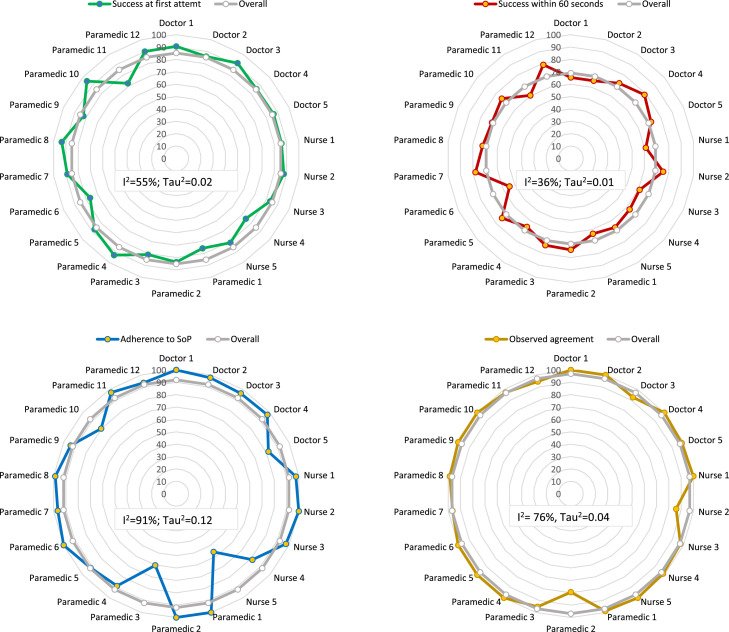
Variability in performance of conducting pulse oximetry assessments by individual IMCI service-providers, presented in percentage. The heterogeneity statistics I^2^ (i.e. proportion of heterogeneity between estimates that is not due to chance) and Tau^2^ (i.e. the magnitude of the heterogeneity) were significant at 5% level of significance.

Regarding performance by individual IMCI service-providers, we observed minimum variability in successfully conducting pulse oximetry assessment in one attempt (I^2^=55%; Tau^2^=0.02), within one minute (I^2^=36%; Tau^2^=0.01), SoP-adherence (I^2^=91%; Tau^2^=0.12) and agreement (I^2^=76%, Tau^2^=0.04) (Figure 3).

### Performance time for conducting pulse oximetry assessment

**Figure 4:**
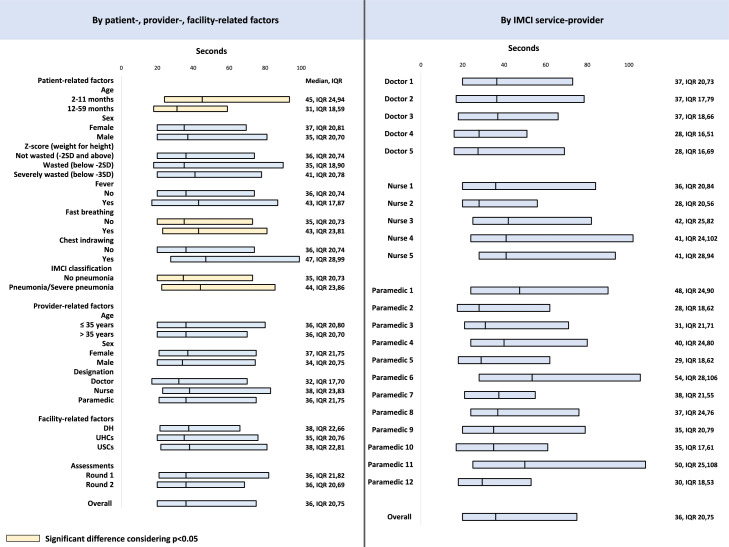
Performance time for conducting pulse oximetry assessment by patient-, provider-, and facility-related factors and by individual IMCI service-providers, presented in median seconds with IQR.

The median performance time was 36 seconds (IQR 20,75) (Figure 4). The performance time was significantly (*p* < 0.01) longer among younger children (aged 2-11 months; 44s, IQR 22,78) than older children (30s, IQR 18,53). Similarly, it took significantly (*p* < 0.01) more time among children classified as pneumonia/severe-pneumonia (41s, IQR 22,70) than those who were classified as no-pneumonia (32s, IQR 20,62). There was wide variability among IMCI service-providers regarding the timing (Pearson chi^2^= 38.0240; *p* < 0.01).

### Device-, patient-, environment- and user-related barriers and challenges faced by the IMCI service-providers

**Figure 5:**
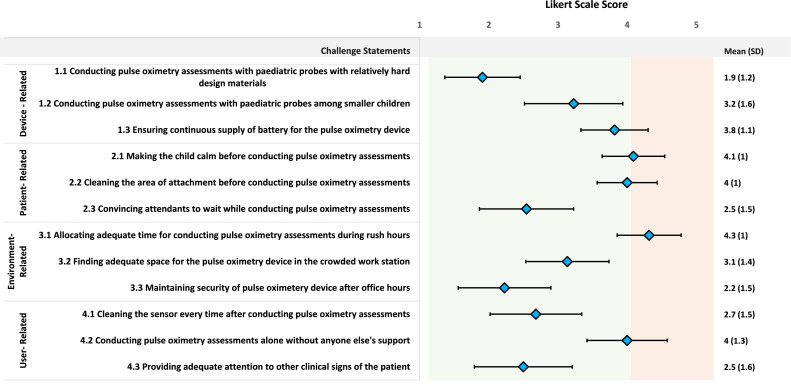
Device-, patient-, environment- and user-related barriers and challenges faced by the IMCI service-providers during pulse oximetry assessments, presented in mean score with SD based on a five-point Likert Scale, N=22. The light green shaded region indicates lower level of challenges and light red shaded region indicates higher level of challenges in in performing pulse oximetry assessments.

Figure 5 presents the barriers and challenges (appropriateness-experience) reported by the IMCI service-providers while conducting pulse oximetry assessments based on a five-point Likert scale. Regarding patient-related factors, the highest (mean 4.1, 95% CI 3.6, 4.5) challenge was faced in making the child calm before conducting pulse oximetry assessments, which was closely followed by cleaning the area of attachment before placing the probe (mean 4.0, 95% CI 3.6, 4.4). Regarding environment-related factors, providers reported a high level of challenge in allocating adequate time for pulse oximetry during the period of peak service use (mean 4.3, 95% CI 3.9, 4.8) and performing pulse oximetry alone (mean 4.0, 95% CI 3.4, 4.6).

### Agreement between IMCI service-providers and study appointed nurses using prevalence-adjusted and bias-adjusted kappa (PABAK)

The IMCI service-providers maintained optimum IPC practice during 72% (95% CI 70-74) of assessments (details-SM16). This was significantly better among paramedics (AOR 2.6, 95% CI 1.8, 3.8) and nurses (AOR 1.8, 95% CI 1.2, 2.7) compared to doctors providing IMCI services, and in sub-district hospitals (AOR 20.3, 95% CI 13.1, 31.6) and health centres (AOR 7.0, 95% CI 4.1, 11.7) compared to the district hospital. It was also substantially improved in round-2 (AOR 4.8, 95% CI 3.7, 6.3).

**Figure 6:**
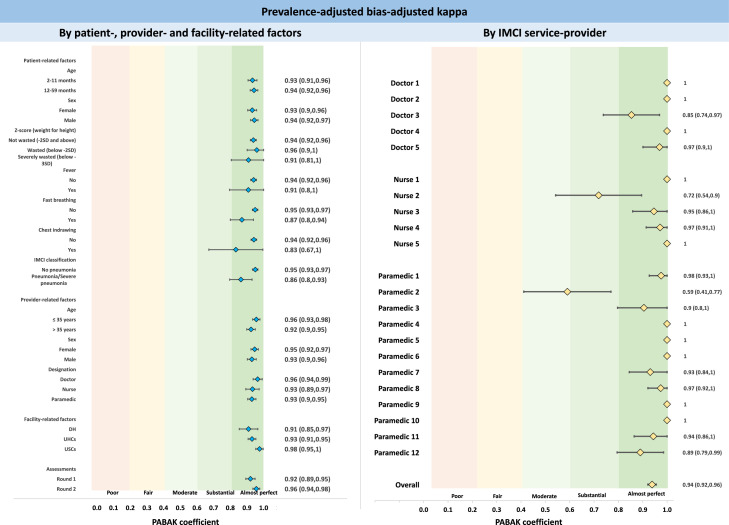
Agreement between IMCI service-providers and study appointed nurses using prevalence-adjusted and bias-adjusted kappa (PABAK) - by patient-related, provider-related and facility-related factors and by individual IMCI service-providers, presented as coefficient and 95% CI. The light red and yellow shaded regions indicate lower agreement PABAK score and the green shaded regions indicate higher agreement PABAK score.

Figure 6 presents the agreement between IMCI service-providers and study appointed nurses using prevalence-adjusted and bias-adjusted kappa (PABAK). The overall PABAK was 0.94; a very high level of agreement. We did not observe significant variation by patient-, provider-, and facility-related factors and by individual IMCI service-providers.

## Discussion

Pulse oximetry has enormous potential for maximising the impact of IMCI in LMIC settings with high burdens of childhood pneumonia and hypoxaemia.^8^^,^^13^ This is one of the novel studies which adopted a health system strengthening approach and district model of implementation. To the best of our knowledge, this is the first study to report most of the WHO implementation outcome variables which include adoption, feasibility, fidelity, appropriateness, acceptability and sustainability. We observed that IMCI service-providers almost universally conducted pulse oximetry assessments on almost all eligible children, and can successfully measure SpO_2_ with minimum attempts, within a relatively short time and without major challenges. Their assessments were accurate, and performance was sustained or generally improved between two rounds. Also, caregivers were very positive towards introducing this technology for assessing their children.

Understanding the adoption and feasibility of introducing a new technology/device into routine systems is crucial to inform policy and programme.^20^ We observed high-level of use, success and usability in conducting pulse oximetry assessment by IMCI service-providers. Although there are some differences in the context, case-mix and assessor characteristics, our findings are comparable to studies conducted in Ethiopia, Malawi, UK, Bangladesh and Pakistan.^12^^,^^13^^,^^24^^,^^25^ These findings from diverse contexts reinforce the likelihood of adoption and feasibility of introducing pulse oximetry in resource-constrained outpatient settings. We have undertaken an inclusive approach of involving stakeholders, including IMCI service-providers, in various stages of planning and implementation. We have also ensured their continuous engagement through training, support supervision and performance appraisal workshops. This stakeholder engagement process may have contributed to the sense of ownership and acquisition of necessary skills, potentially explaining the encouraging findings related to adoption and feasibility.^18^ The first wave of the COVID-19 pandemic started a few months before the round-1 assessments. Although the pandemic created barriers in delivering many essential health services, it positively influenced the awareness of health care providers regarding hypoxaemia and attitude towards pulse oximetry, and may have contributed as an unexpected enabler in this study.^26^^,^^27^

Fidelity of implementation is integrally linked to achieving the intended benefit of an intervention.^20^ In our study, we observed a high-level of SoP-adherence by IMCI service-providers, similar to a study in rural Ethiopia.^24^ A systematic review reported that communicating through multiple approaches can significantly improve SoP-adherence among healthcare providers.^28^ We also adopted multiple approaches, such as emphasising the importance of calming the child and correct probe placement, along with ensuring availability of job-aids/visual aids in IMCI consultation rooms, which may have promoted such practice. However, we observed some issues related to IPC practices. Although probe cleaning procedures were not associated with successfully conducting pulse oximetry assessments, they represent an indispensable element of infection control, particularly in the COVID-19 context.^29^

Regarding identifying hypoxaemia through pulse oximetry, we observed a high-level of agreement between IMCI service-providers and study nurses. According to the WHO, fast breathing/chest-in-drawing as the only sign of pneumonia can be treated through outpatient management.^10^ However, around one in 10 such cases experience treatment failure, suggesting the presence of some 'severe cases' among apparently 'non-severe' cases of pneumonia.^30^ Hypoxemia is one of the strongest predictors of adverse clinical outcomes, and its accurate identification can help reduce the misclassification of non-severe pneumonia.^7^^,^^31^^,^^32^ The high-level of agreement should give confidence to policymakers and families regarding the accuracy of SpO_2_ measurement by minimally trained providers and thus their ability to identify the severe cases, even in routine settings.

We did not find any significant effect of provider- or facility-related factors on adoption and feasibility, suggesting that, with health systems inputs, such as training and post-training follow-up support through supervision and performance appraisal, different types of IMCI service-providers can achieve necessary pulse oximetry skills in all kinds of facilities. Regarding patient-related factors, similar to a multi-country trial, we observed that success and usability were significantly poorer among younger children, with performance time decreasing with increasing age.^25^ The providers interviewed in our study and several other studies also reported facing difficulties in conducting pulse oximetry among younger and smaller children.^11^^,^^33^ Pulse oximetry readings are significantly affected by patient movement (motion artefacts) and the appropriateness of the probe size.^34^ Younger children are expected to be more restless during pulse oximetry. Although we used Masimo paediatric probes of same size in this study, the size, design and material used for these probes may need specific adaptations, especially in settings with high burdens of both pneumonia and malnutrition, which is the case for Bangladesh.^25^ Around a third of the children are underweight or stunted in Bangladesh.^35^ For the undernourished children it requires further adaptation for the best fit of the probes. We also found that success in one attempt was higher among male children, despite there being no notable differences in the clinical presentation of the children by sex. Although this requires more exploration, some studies have reported the presence of implicit gender bias among healthcare professionals while rendering care in routine settings.^36^

With regard to fidelity, we observed that the male and younger IMCI service-providers adhered to SoPs more than their counterparts. This may be because they have more access to new technology/devices in such contexts.^37^ Another reason could be that the average year of experience was higher among male providers. However, it requires further exploration and understanding. On facility-related factors, we observed that IMCI service-providers at higher-level facilities are more likely to adhere to SoPs. Service-providers in the district hospital usually receive more monitoring and supervision from the national and district health managers. These variations in programme inputs may explain this difference.

Pulse oximetry performance was better in round-2 assessments across most indicators. This strengthens the notion of sustainability of pulse oximetry practice and highlights the importance of allowing an intervention to mature and reach optimum adoption and efficiency. Post-training follow-up support provided through supportive supervision and performance appraisal workshops (after round-1 assessments) possibly played a catalytic role in sustaining and improving the performance over time. The performance of a few providers was somewhat poorer than others, emphasising the importance of identifying weaker performers during the initial training and providing follow-up support through targeted monitoring and supervision.

There are several barriers and challenges to performing pulse oximetry in real-life settings. In our study, IMCI service-providers reported difficulties during peak hours of attendance at health facilities. In Bangladesh, most IMCI services are provided through union-level health centres (> # 3500) and sub-district hospitals (∼# 482), which have a relatively lower patient load than district hospitals (# 62). Hence, the peak-hour related challenges are primarily a concern for district hospitals and some high-volume sub-district hospitals. The IMCI service-providers also reported facing major difficulties in keeping the child calm during pulse oximetry, which is mainly a concern for high volume facilities. Pulse oximetry devices with advanced Signal Extraction Technology (SET) require a shorter performance time, even in challenging conditions such as patient movement and low perfusion.^34^^,^^38^ Although relatively more expensive ($300-500), it will require less than USD 250,000 to procure SET pulse oximetry devices for all high-volume IMCI consultation rooms in Bangladesh. We believe that this is affordable for a country like Bangladesh, as the overall national IMCI-programme under the Maternal, Neonatal, Child & Adolescent Health (MNC&AH) Operation Plan of Bangladesh has a total of around 50 million USD allocated for its activities. We believe, it is worth the investment to avert preventable pneumonia deaths by 2025.^39^ In addition to enhancing the capacity of IMCI service-providers through training and distributing toys to divert the attention of the child, active involvement of the caregivers has been shown to be effective in reducing the movement of the child during the procedure.^11^

We conceptualised this study based on the WHO framework and implementation outcome variables.^20^ Although we contextualised the research questions by involving national- and local-level stakeholders, our reliance on WHO's implementation outcome variables limited the opportunities of capturing unexpected drivers and unintended consequences of pulse oximetry introduction in these settings.^18^ Moreover, we acknowledge that the time interval between the two rounds of assessments were not sufficient to allow for firm conclusions to be made regarding the sustainability. Furthermore, the COVID-19 pandemic may have confounded some of our findings as it significantly impacted Bangladesh's health systems awareness, attitude, readiness, response, and practices related to oxygen security. Similarly, the capacity, experience and positionality of icddr,b as an implementation facilitation partner was a strong enabler and a potential confounder in this demonstration.^17^^,^^40^

We acknowledge the fact that we did not randomly select Kushtia as our demonstration site. Therefore, the result may not be applicable for the whole country. In Bangladesh, there are geographical variations which have impact on access to health care as well as service availability and readiness regarding various health services. Kushtia predominantly belongs to the plain land which is not representative of the hard-to-reach areas. Based on our findings of this study, pulse oximetry can be introduced in most parts of Bangladesh except the hard-to-reach areas. We also acknowledge that we have selected a district with reasonably well functioning IMCI services. The results would have been more generalisable if we could have included one low performing district which we could not include due to implementation cost challenges.

We employed the same team in both rounds of assessment and used a specially designed and tested android-app for capturing multiple events with time-stamps through structured-observations.^21^ These measures, we believe, reduced observation and measurement bias, increasing the validity and reliability of measurements, especially the performance time recording. While the presence of study nurses as independent observers may have introduced a surveillance bias, spending considerable time in building rapport with the IMCI service-providers and observing each of them for 3-7 days are expected to minimise this bias (i.e., Hawthorne effect).^41^ Finally, the hospitals had set-up temporary fever-clinics during the pandemic, which screened and redirected all symptomatic patients to dedicated COVID-19 services. It changed the overall case-mix of children receiving IMCI services, which may explain the relatively smaller number of hypoxaemia cases identified during the assessment period. Lastly, we acknowledge that the data collection tools used in this study for structured-observation, re-assessment, exit-interview and IMCI service-provider interview were not validated. Since we were conducting a novel study based on the WHO recommended implementation outcome variables, we did not find any validated tools. Therefore, we conducted extensive desk review to construct the data collection tools. We also conducted qualitative explorations for understanding the major issues related to acceptance, barriers and challenges, which helped us preparing the exit-interview and IMCI service-provider interview tools. We conducted field-testing and pretesting of each tool before finalisation. We believe that these measures have contributed in improving the content and construct validity of tools that were used in this study.

This implementation research study demonstrated the feasibility of introducing and integrating pulse oximetry in routine IMCI services in Bangladesh, adopting a district implementation model and using a health system strengthening approach. We are confident in our conclusion as we saw evidence of the programme maturing and improving over time and almost no influence of patient-, provider- and facilities-related factors on SpO_2_ measurement, except that it was poorer among younger children, and a few providers performed the procedure less effectively. Introducing SET pulse oximetry devices in high-volume facilities can help overcome the challenges associated with motion artefacts, particularly among younger children. Special emphasis can be given to identifying providers who would benefit from targeted monitoring and follow-up support. Routine programmes will need to emphasise adherence to SoPs and IPC practices in training and routine monitoring. The policymakers can capitalise on these encouraging findings and ratify the integration of pulse oximetry with respiratory rate assessment in routine outpatient settings. Moreover, we also highlight the importance of connecting pulse oximetry to a functioning oxygen delivery system and ensuring timely, adequate and appropriate oxygenation. We recommend future studies to assess the feasibility of pulse oximetry at community settings of Bangladesh. We also recommend further study on post-implementation follow-up to assess the sustainability and research looking at feasibility, effectiveness and impact over wider interval. The experience gained through this demonstration and the learning synthesised in this implementation research can help inform evidence-based decisions relating to the introduction and scale-up of pulse oximetry in Bangladesh and other LMICs, as these countries aim to avert all preventable deaths due to childhood pneumonia mortality by 2025.

## Contributors

AER conceptualised and developed the original draft of the manuscript as a first author. SEA and HC provided their guidance and feedback to AER at every stage as joint senior authors. They also supported AER in fund acquisition. HN and DD supervised AER in conceptualisation of the research idea. SA assisted AER in data curation and software development for data collection. AER, SA and ATH contributed to formal analysis for the manuscript. JP and SC helped in conceptualising the methodology of the study. SJ, TM, AAU and MZHS supported in project administration, and reviewed the draft manuscript. MSI, MJI, SA, HMS and AS from IMCI programme and SA, GB, AEK and MK from other development partners controbuted in finalisng the study desing and reviewed and edited the draft manuscript. AA and MJC contributed in interpretation of the findings and drafting the manuscript. All authors reviewed and shared feedback on the manuscript and approved the final draft. AER, SA and ATH had access to all data and take full responsibility for its integrity and the accuracy of the analyses. AER had final responsibility for the decision to submit for publication.

## Data sharing statement

The data can be shared upon reasonable request to the corresponding author.

## Declaration of interests

SEA and HN from the authors have declared that this research work received grant from NIHR Global Health Research Unit on Respiratory Health (RESPIRE) through the University of Edinburgh, UK, using UK Aid from the UK Government. HN declared receiving grants from innovative Medicine Initiative and WHO in past 36 months; consulting fees from Sanofi Pasteur, WHO and Bill and Melinda Gates Foundation; and payment or honoraria from AbbVie; and Participation on a Data Safety Monitoring Board or Advisory Board in Janseen, Reviral, AbbVie. All the other authors report no conflicts of interest.

## Appendix Supplementary materials

Image, application 1

Image, application 2

Image, application 3
