# Supplementary material for: Introducing pulse oximetry for outpatient management of childhood pneumonia: An implementation research adopting a district implementation model in selected rural facilities in Bangladesh
Source: eClinicalMedicine. 2022 Jun 29;50:101511. doi: 10.1016/j.eclinm.2022.101511 (PMC9251564; doi:10.1016/j.eclinm.2022.101511)
Supplement: Supplementary file 1 [file mmc1.docx]

**Caption for supplementary material**

**Supplementary material 1: Study site and health facilities.**

Caption: Study site and health facilities selected for introducing pulse oximetry in Kushtia, Bangladesh.

**Supplementary material 2: Type, staffing pattern, service availability and IMCI service utilisation of health facilities.**

Caption: Type, staffing pattern, service availability and IMCI service utilisation of health facilities selected for introducing pulse oximetry.

**Supplementary material 3: Important milestones**.

Caption: Important milestones for the design, development, and demonstration of the district implementation model.

**Supplementary material 4: Primary and secondary research questions and analysis plan.**

Caption: Primary and secondary research questions and analysis plan based on paper objectives.

**Supplementary material 5: Background characteristics**.

Caption: Background characteristics of the data collection team.

**Supplementary material 6: Snapshots.**

Caption: Snapshots of the survey app.

**Supplementary material 7: Normality distribution of timing.**

Caption: Normality distribution of timing using the Shapiro–Wilk test.

**Supplementary material 8: Background characteristics of the IMCI service-providers.**

Caption: Background characteristics of the IMCI service-providers by rounds of assessment.

**Supplementary material 9: Background characteristics of the children presenting with cough/difficulty-in-breathing.**

Caption: Background characteristics of the children presenting with cough/difficulty-in-breathing by rounds of assessment.

**Supplementary material 10: Number of children assessed by background characteristics of the IMCI services-providers.**

Caption: Number of children assessed by background characteristics of the IMCI services-providers by rounds of assessment.

**Supplementary material 11: Number of assessments by IMCI service-providers.**

Caption: Number of assessments by IMCI service-providers, by round.

**Supplementary material 12: Influence of several patient-, provider-, and facility-related factors on successfully conducting pulse oximetry assessments at the first attempt by IMCI services-providers**.

Caption: Influence of several patient-, provider-, and facility-related factors on successfully conducting pulse oximetry assessments at the first attempt by IMCI services-providers presented in adjusted odds ratios, N=1680.

**Supplementary material 13: Influence of several patient-, provider-, and facility-related factors on successfully conducting pulse oximetry assessments within one minute by IMCI services-providers.**

Caption: Influence of several patient-, provider-, and facility-related factors on successfully conducting pulse oximetry assessments within one minute by IMCI services-providers, presented in adjusted odds ratios, N=1680.

**Supplementary material 14: Influence of several patient-, provider-, and facility-related factors on adhering to SoPs while conducting pulse oximetry assessments by IMCI services-providers.**

Caption: Influence of several patient-, provider-, and facility-related factors on adhering to SoPs while conducting pulse oximetry assessments by IMCI services-providers presented in adjusted odds ratios, N=1680.

**Supplementary material 15: Influence of several patient-, provider-, and facility-related factors on agreement of identifying hypoxaemia through pulse oximetry between IMCI services-providers and study nurses.**

Caption: Influence of several patient-, provider-, and facility-related factors on agreement of identifying hypoxaemia through pulse oximetry between IMCI services-providers and study nurses, presented in adjusted odds ratios, N=1680.

**Supplementary material 16: Influence of several patient-, provider-, and facility-related factors on optimum cleanliness practices by IMCI services-providers.**

Caption: Influence of several patient-, provider-, and facility-related factors on optimum cleanliness practices by IMCI services-providers, presented in adjusted odds ratios, N=1680.

**Supplementary material 17: Summary of findings on the secondary research questions.**

Caption: Summary of findings on the secondary research questions based on paper objectives.
