# Supplementary material for: Introducing pulse oximetry for outpatient management of childhood pneumonia: An implementation research adopting a district implementation model in selected rural facilities in Bangladesh
Source: eClinicalMedicine. 2022 Jun 29;50:101511. doi: 10.1016/j.eclinm.2022.101511 (PMC9251564; doi:10.1016/j.eclinm.2022.101511)
Supplement: Supplementary file 3 [file mmc3.docx]

**Supplementary material 1: Study site and health facilities.**

Caption: Study site and health facilities selected for introducing pulse oximetry in Kushtia, Bangladesh.


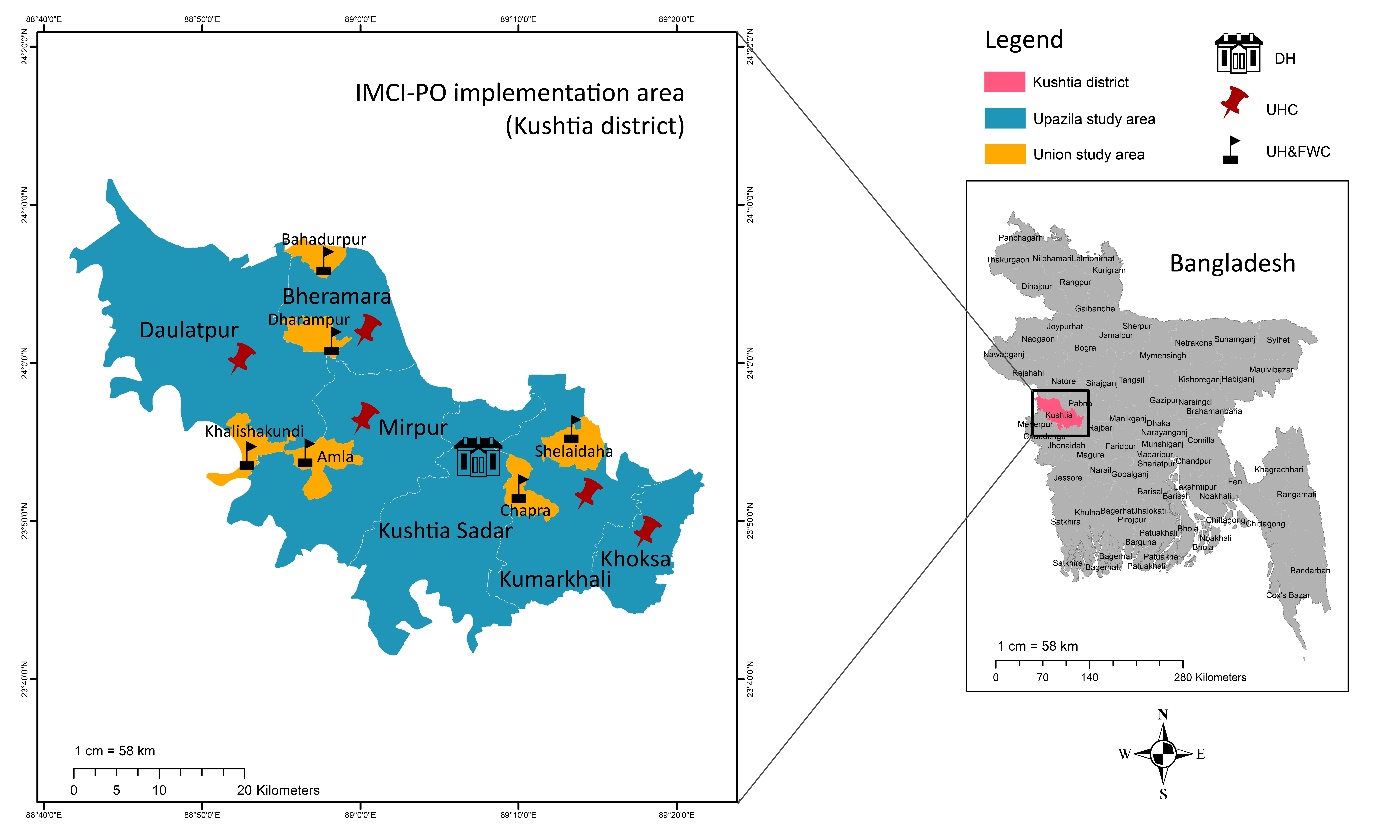


**Supplementary material 2: Type, staffing pattern, service availability and IMCI service utilisation of health facilities.**

Caption: Type, staffing pattern, service availability and IMCI service utilisation of health facilities selected for introducing pulse oximetry.

| **Name** | **Type of Health Facility** | **Indoor service** | **Separate paediatric indoor** | **Emergency service** | **Outdoor service** | **IMCI service** | **Separate IMCI corner** | **IMCI utilisation** | | | | | | | | |
| --- | --- | --- | --- | --- | --- | --- | --- | --- | --- | --- | --- | --- | --- | --- | --- | --- |
|  |  |  |  |  |  |  |  | **All children aged 2-59 months** | | | **Severe Pneumonia** | | | **Pneumonia** | | |
|  |  |  |  |  |  |  |  | **2019** | **2020** | **2021** | **2019** | **2020** | **2021** | **2019** | **2020** | **2021** |
| Kushtia DH | District Hospital | Yes, 250-bedded | Available | Available | Available | Available | Available | 20,294 | 12,299 | 13,656 | 3,218 | 697 | 52 | 3,888 | 744 | 408 |
| Kumarkhali UHC | Upazila Health Complex | Yes, 50-bedded | Not Available | Available | Available | Available | Available | 18,495 | 6,636 | 8,891 | 367 | 227 | 267 | 655 | 309 | 307 |
| Bheramara UHC | Upazila Health Complex | Yes, 50-bedded | Not Available | Available | Available | Available | Available | 5,066 | 3,150 | 5,355 | 32 | 2 | 8 | 877 | 379 | 443 |
| Mirpur UHC | Upazila Health Complex | Yes, 50-bedded | Not Available | Available | Available | Available | Available | 9,072 | 4,386 | 4,610 | 16 | 4 | 0 | 16 | 1 | 11 |
| Khoksa UHC | Upazila Health Complex | Yes, 50-bedded | Not Available | Available | Available | Available | Available | 10,820 | 6,578 | 4,815 | 128 | 31 | 1 | 3,039 | 610 | 42 |
| Daulatpur UHC | Upazila Health Complex | Yes, 50-bedded | Not Available | Available | Available | Available | Available | 6,349 | 4,262 | 3,465 | 505 | 313 | 171 | 672 | 654 | 293 |
| Shelaidaha USC | Union Sub-Centres | Not Available | Not Applicable | Not Available | Available | Available | Not Available | 1,129 | 1,913 | 750 | 0 | 0 | 36 | 151 | 218 | 77 |
| Saota USC | Union Sub-Centres | Not Available | Not Applicable | Not Available | Available | Available | Not Available | 1,274 | 932 | 798 | 0 | 0 | 57 | 30 | 92 | 148 |
| Bahadurpur USC | Union Sub-Centres | Not Available | Not Applicable | Not Available | Available | Available | Not Available | 1,469 | 1,009 | 1,103 | 24 | 178 | 17 | 623 | 193 | 63 |
| Dharampur USC | Union Sub-Centres | Not Available | Not Applicable | Not Available | Available | Available | Not Available | 2,784 | 877 | 955 | 0 | 0 | 0 | 1,094 | 311 | 0 |
| Amla USC | Union Sub-Centres | Not Available | Not Applicable | Not Available | Available | Available | Not Available | 490 | 888 | 618 | 0 | 0 | 0 | 0 | 40 | 214 |
| Khalishakundi USC | Union Sub-Centres | Not Available | Not Applicable | Not Available | Available | Available | Not Available | 444 | 299 | 747 | 12 | 0 | 0 | 407 | 247 | 3 |

**Supplementary material 3: Important milestones**.

Caption: Important milestones for the design, development, and demonstration of the district implementation model.


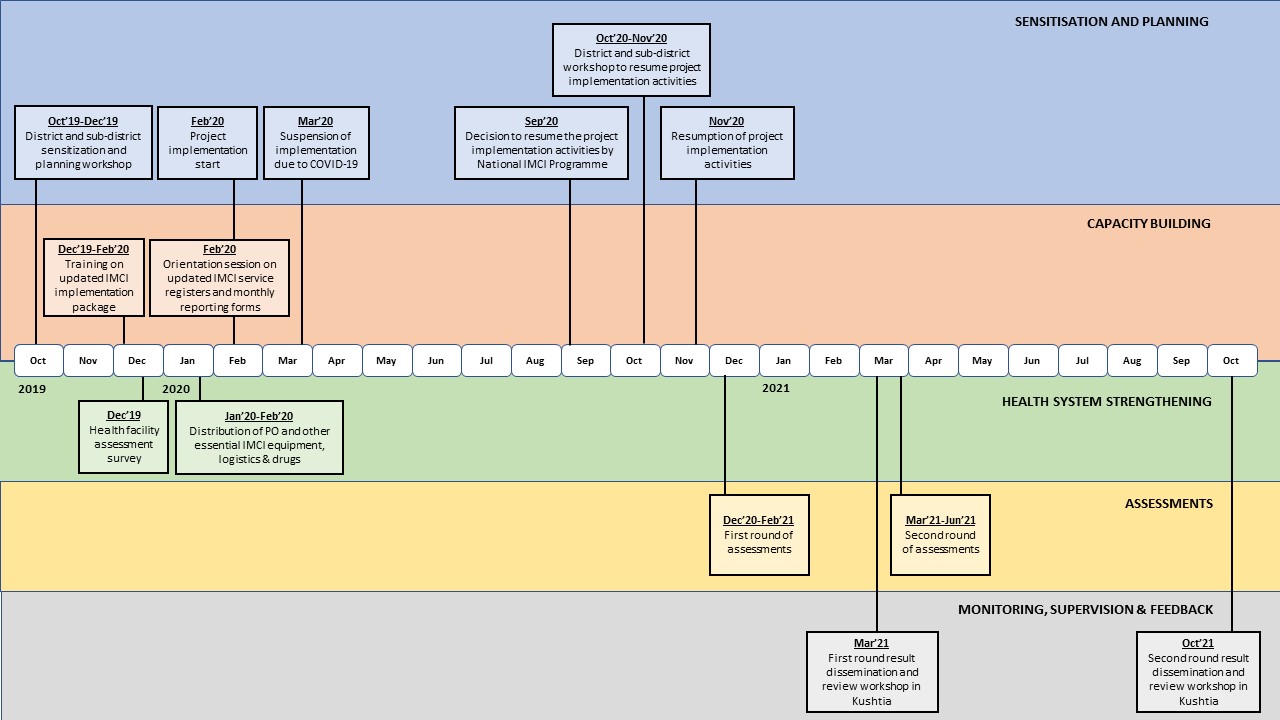


**Supplementary material 4: Primary and secondary research questions and analysis plan.**

Caption: Primary and secondary research questions and analysis plan based on paper objectives.

| **WHO’s  Implementation Outcome Variable** | **Research questions for performing pulse oximetry in routine outpatient settings** | **Statistical test** | **Rationale and details** |
| --- | --- | --- | --- |
|  | **Primary Research Questions** |  |  |
| 1. **Adoption** | - 1. Use: Do IMCI service-providers conduct pulse oximetry assessments? | percentage | Rates presented using percentage. |
| 1. **Feasibility** | - 1. Success: Can IMCI service- providers successfully conduct pulse oximetry assessments? | Percentage with 95% Confidence Interval | Rates presented using percentage and confidence interval. |
|  | - 1. Usability by attempt: Can IMCI service-providers successfully conduct pulse oximetry assessments at the first attempt? | Percentage with 95% Confidence Interval | Rates presented using percentage and confidence interval. |
|  | - 1. Usability by time: Can IMCI service-providers successfully conduct pulse oximetry assessments within one minute? | Percentage with 95% Confidence Interval | Rates presented using percentage and confidence interval. |
| 1. **Fidelity** | - 1. Adherence: Do IMCI service-providers follow Standard Operating Procedure (SoP) while conducting pulse oximetry assessments? | Percentage with 95% Confidence Interval | Rates presented using percentage and confidence interval. |
|  | - 1. Agreement: Can IMCI service-providers identify hypoxaemia through pulse oximetry? | Percentage with 95% Confidence Interval | Rates presented using percentage and confidence interval. |
| 1. **Appropriateness** | - 1. Experience: Do IMCI service- providers conduct pulse oximetry assessments with reasonably low barriers and challenges? | Mean score from 5-point Likert scale with 95% confidence interval | Mean score with confidence interval reported to present the barriers and challenges of performing pulse oximetry to present the central tendency and dispersion of the likert statement scores. |
| 1. **Acceptability** | - 1. Usefulness: Do IMCI service-providers perceive pulse oximetry as useful? | Percentage with 95% Confidence Interval | Rates presented using percentage and confidence interval. |
|  | - 1. Importance: Do the caregivers perceive pulse oximetry as important? | Percentage with 95% Confidence Interval | Rates presented using percentage and confidence interval. |
|  | - 1. Satisfaction: Are the caregivers satisfied with pulse oximetry introduction in routine IMCI services? | Percentage with 95% Confidence Interval | Rates presented using percentage and confidence interval. |
| 1. Sustainability | - 1. Sustainability: Does the pulse oximetry performance of IMCI service-providers sustain over time (rounds)? | Percentage with 95% Confidence Interval | Rates presented using percentage and confidence interval. |
|  | **Secondary Research Questions** |  |  |
| **Feasibility** | - 1. Can IMCI service-providers successfully conduct pulse oximetry assessments in two attempts? | Percentage with 95% Confidence Interval | Rates presented using percentage and confidence interval. |
|  | - 1. Can IMCI service-providers successfully conduct pulse oximetry in three attempts? | Percentage with 95% Confidence Interval | Rates presented using percentage and confidence interval. |
|  | - 1. Do various patient-, provider- and facility-related factors influence successfully conducting pulse oximetry assessments at the first attempt? | Percentage with 95% Confidence Interval | We assume correlation within the assessments by each assessor. Therefore, GEE has been performed for accounting for this correlation in analysis. Wald statistics used for checking adequacy of model. |
|  | - 1. Is there any variation in successfully conducting pulse oximetry assessments at the first attempt by individual IMCI service-providers? | Proportion with heterogeneity statistics across individual assessors (i.e. I2 an Tau2 statistics) | We assumed random variation within assessments by each assessor. Heterogeneity statistics I^2^ was used to report the proportion of between assessor variability which is not due to chance and Tau^2^ has been reported to report the actual between assessor variability. |
|  | - 1. Can IMCI service-providers successfully conduct pulse oximetry assessment in three minutes? | Percentage with 95% Confidence Interval | Rates presented using percentage and confidence interval. |
|  | - 1. Can IMCI service-providers successfully conduct pulse oximetry assessment in five minutes? | Percentage with 95% Confidence Interval | Rates presented using percentage and confidence interval. |
|  | - 1. Do various patient-, provider-, and facility-related factors influence successfully conducting pulse oximetry assessments within one minute? | Generalized Estimating Equation reporting odds ratios with 95% confidence interval | We assume correlation within the assessments by each assessor. Therefore, GEE has been performed for accounting for this correlation in analysis. Wald statistics used for checking adequacy of model. |
|  | - 1. Is there any variation in successfully conducting pulse oximetry assessments within one minute by individual IMCI service-providers? | Proportion with heterogeneity statistics across individual assessors (i.e. I2 an Tau2 statistics) | We assumed random variation within assessments by each assessor. Heterogeneity statistics I^2^ was used to report the proportion of between assessor variability which is not due to chance and Tau^2^ has been reported to report the actual between assessor variability. |
|  | - 1. What is the performance time for successfully conducting pulse oximetry assessments? | Median with Interquartile range (IQR) | Shapiro-Wilk test presented the non-normality of the distribution of the time taken for measurement of SpO_2_ . Hence, median times were reported with IQR. |
|  | - 1. Do various patient-, provider-, and facility-related factors influence performance time for successfully conducting pulse oximetry assessments? | Mood’s Median Test | Shapiro-Wilk test presented the non-normality of the distribution of the time taken for measurement of SpO_2_. Hence, median times were reported with IQR and non-parametric equality of median tests have been performed. |
|  | - 1. Is there any variation in performance time for successfully conducting pulse oximetry assessments by individual IMCI service-providers? | Mood’s Median Test | Shapiro-Wilk test presented the non-normality of the distribution of the time taken for measurement of SpO_2_. Hence, median times were reported with IQR and non-parametric equality of median tests have been performed. |
| **Fidelity** | - 1. Do IMCI service-providers ensure the baby was calm before conducting pulse oximetry assessments? | Percentage with 95% Confidence Interval | Rates presented using percentage and confidence interval. |
|  | - 1. Do they place the probe appropriately before conducting pulse oximetry assessments? | Percentage with 95% Confidence Interval | Rates presented using percentage and confidence interval. |
|  | - 1. Do various patient-, provider-, and facility-related factors influence adhering to Standard Operating Procedure (SoP) while conducting pulse oximetry assessments? | Generalized Estimating Equation reporting odds ratios with 95% confidence interval | We assume correlation within the assessments by each assessor. Therefore, GEE has been performed for accounting for this correlation in analysis. Wald statistics used for checking adequacy of model. |
|  | - 1. Is there any variation in adhering to Standard Operating Procedure (SoP) while conducting pulse oximetry assessment by individual IMCI-service providers? | Proportion with heterogeneity statistics across individual assessors (i.e. I2 an Tau2 statistics) | We assumed random variation within assessments by each assessor. Heterogeneity statistics I^2^ was used to report the proportion of between assessor variability which is not due to chance and Tau^2^ has been reported to report the actual between assessor variability. |
|  | - 1. Do IMCI service-providers adhere to cleanliness practices before conducting pulse oximetry assessments? | Percentage with 95% Confidence Interval | Rates presented using percentage and confidence interval. |
|  | - 1. Do various patient-, provider-, and facility-related factors influence adhering to cleanliness practices while conducting pulse oximetry assessments? | Generalized Estimating Equation reporting odds ratios with 95% confidence interval | We assume correlation within the assessments by each assessor. Therefore, GEE has been performed for accounting for this correlation in analysis. Wald statistics used for checking adequacy of model. |
|  | - 1. Is there any variation in adhering to cleanliness practices while conducting pulse oximetry assessments by individual IMCI service-providers? | Proportion with heterogeneity statistics across individual assessors (i.e. I2 an Tau2 statistics) | We assumed random variation within assessments by each assessor. Heterogeneity statistics I^2^ was used to report the proportion of between assessor variability which is not due to chance and Tau^2^ has been reported to report the actual between assessor variability. |
|  | - 1. Do various patient-, provider-, and facility-related factors influence agreement of hypoxaemia identification (SpO2<94%) thorough pulse oximetry? | Generalized Estimating Equation reporting odds ratios with 95% confidence interval | We assume correlation within the assessments by each assessor. Therefore, GEE has been performed for accounting for this correlation in analysis. Wald statistics used for checking the adequacy of model. |
|  | - 1. Is there any variation in agreement of hypoxaemia identification (SpO2<94%) through pulse oximetry by individual IMCI service-providers? | Proportion with heterogeneity statistics across individual assessors (i.e. I2 and Tau2 statistics) | We assumed random variation within assessments by each assessor. Heterogeneity statistics I^2^ was used to report the proportion of between assessor variability which is not due to chance and Tau^2^ has been reported to report the actual between assessor variability. |

**Supplementary material 5: Background characteristics**.

Caption: Background characteristics of the data collection team.

| **Data collector** | **Data collection type** | **Age (in years)** | **Sex** | **Educational qualification** | **Experience (in years)** |
| --- | --- | --- | --- | --- | --- |
| Nurse 1 | Observation | 24 | Female | Diploma in Nursing & Midwifery | 3 |
| Nurse 2 | Observation | 22 | Female | Diploma in Nursing & Midwifery | 2 |
| Nurse 3 | Re-assessment | 24 | Female | Diploma in Nursing & Midwifery | 2 |
| Nurse 4 | Re-assessment | 23 | Male | Diploma in Nursing & Midwifery | 1 |
| Paramedic 1 | Exit interview | 28 | Male | Diploma in Medical Faculty | 7 |
| Paramedic 2 | Exit interview | 24 | Female | Diploma in Medical Faculty | 6 |
| Data extractor 1 | Data Extraction | 30 | Male | Master of Social Science | 6 |
| Data extractor 2 | Data Extraction | 31 | Male | Master of Arts | 3 |

**Supplementary material 6: Snapshots.**

Caption: Snapshots of the survey app.


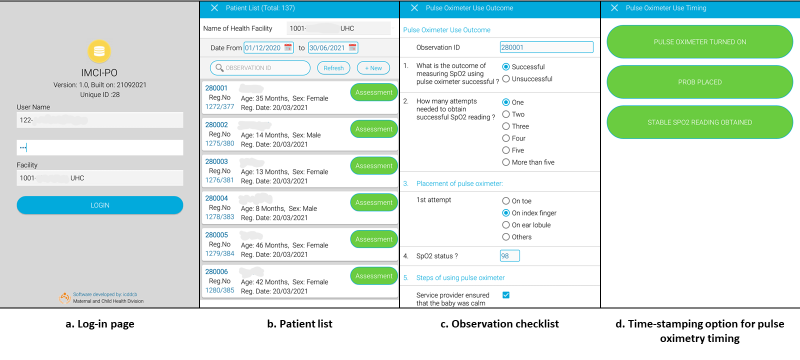


**Supplementary material 7: Normality distribution of timing.**

Caption: Normality distribution of timing using the Shapiro–Wilk test.

| 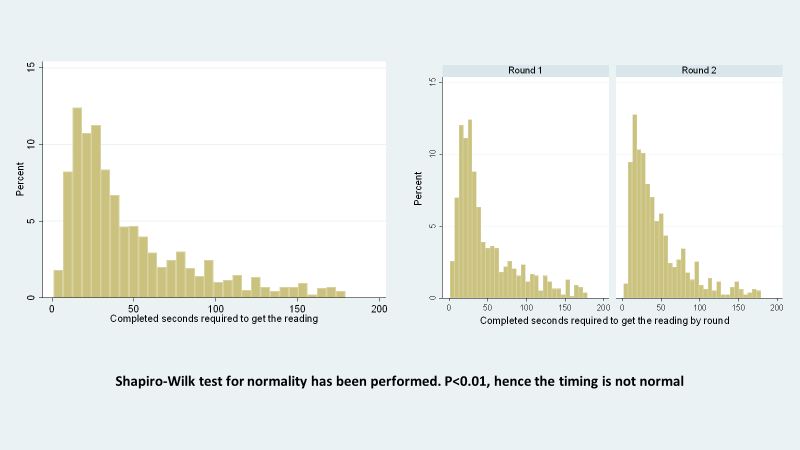 |
| --- |
| 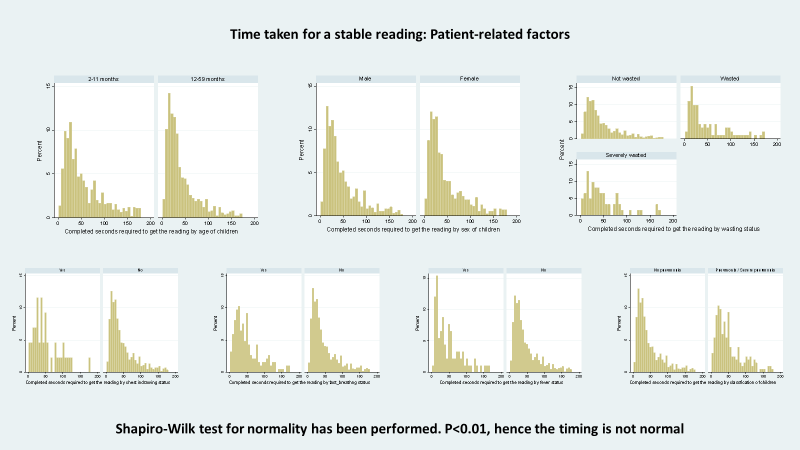 |
| 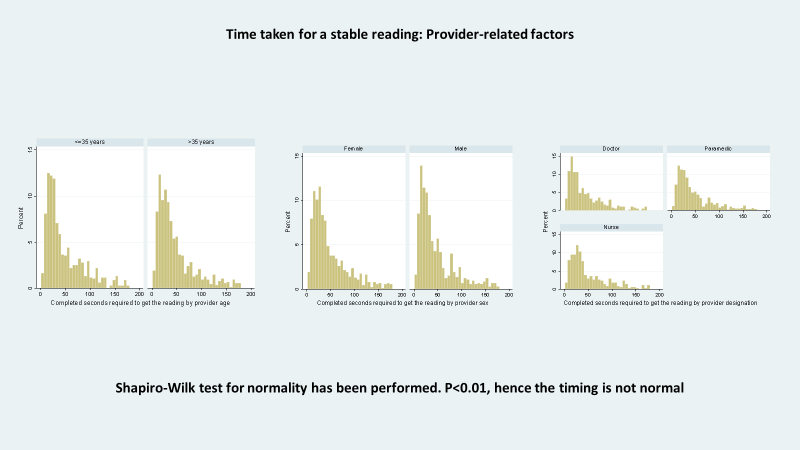 |
| 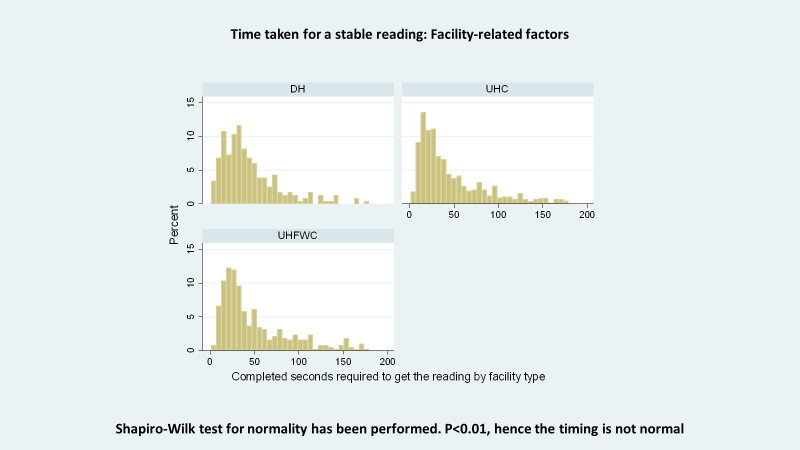 |

**Supplementary material 8: Background characteristics of the IMCI service-providers.**

Caption: Background characteristics of the IMCI service-providers by rounds of assessment.

|  | **Round 1** |  | **Round 2** |  | **All rounds** |  |
| --- | --- | --- | --- | --- | --- | --- |
|  | **n** | **%** | **n** | **%** | **n** | **%** |
| **Service provider characteristics** |  |  |  |  |  |  |
| **Age** |  |  |  |  |  |  |
| **≤ 35 years** | 10 | 46 | 10 | 46 | 10 | 46 |
| **> 35 years** | 12 | 55 | 12 | 55 | 12 | 55 |
| **Sex** |  |  |  |  |  |  |
| **Male** | 10 | 46 | 10 | 46 | 10 | 46 |
| **Female** | 12 | 55 | 12 | 55 | 12 | 55 |
| **Designation** |  |  |  |  |  |  |
| **Doctor** | 5 | 23 | 5 | 23 | 5 | 23 |
| **Nurse** | 5 | 23 | 5 | 23 | 5 | 23 |
| **Paramedic** | 12 | 55 | 12 | 55 | 12 | 55 |
| **Facility type** |  |  |  |  |  |  |
| **District Hospital** | 3 | 14 | 3 | 14 | 3 | 14 |
| **Sub-District Hospital** | 13 | 59 | 13 | 59 | 13 | 59 |
| **Union-level Health Centre** | 6 | 27 | 6 | 27 | 6 | 27 |
| **Total** | 22 | 100 | 22 | 100 | 22 | 100 |

**Supplementary material 9: Background characteristics of the children presenting with cough/difficulty-in-breathing.**

Caption: Background characteristics of the children presenting with cough/difficulty-in-breathing by rounds of assessment.

|  | **Round 1** |  | **Round 2** |  | **All rounds** |  |
| --- | --- | --- | --- | --- | --- | --- |
|  | **n** | **%** | **n** | **%** | **n** | **%** |
| **Patient characteristics** |  |  |  |  |  |  |
| **Age** |  |  |  |  |  |  |
| **2-11 months** | 355 | 42 | 374 | 45 | 729 | 43 |
| **12-59 months** | 500 | 59 | 451 | 55 | 951 | 57 |
| **Sex** |  |  |  |  |  |  |
| **Male** | 455 | 53 | 453 | 55 | 908 | 54 |
| **Female** | 400 | 47 | 372 | 45 | 772 | 46 |
| **Z-score (weight for height)** |  |  |  |  |  |  |
| **Not wasted (-2SD and above)** | 775 | 92 | 725 | 88 | 1500 | 90 |
| **Wasted (below -2SD)** | 36 | 4 | 62 | 8 | 98 | 6 |
| **Severely wasted (below -3SD)** | 30 | 4 | 36 | 4 | 66 | 4 |
| **Missing** | 14 |  | 2 |  | 16 |  |
| **Fever** |  |  |  |  |  |  |
| **No** | 811 | 95 | 765 | 93 | 1576 | 94 |
| **Yes** | 44 | 5 | 60 | 7 | 104 | 6 |
| **Fast breathing** |  |  |  |  |  |  |
| **No** | 734 | 88 | 726 | 88 | 1460 | 88 |
| **Yes** | 102 | 12 | 97 | 12 | 199 | 12 |
| **Missing** | 19 |  | 2 |  | 21 |  |
| **Chest indrawing** |  |  |  |  |  |  |
| **No** | 823 | 98 | 793 | 96 | 1616 | 97 |
| **Yes** | 18 | 2 | 30 | 4 | 48 | 3 |
| **Missing** | 14 |  | 2 |  | 16 |  |
| **IMCI classification** |  |  |  |  |  |  |
| **No pneumonia** | 727 | 86 | 717 | 87 | 1444 | 87 |
| **Pneumonia/Severe pneumonia** | 114 | 14 | 106 | 13 | 220 | 13 |
| **Missing** | 14 |  | 2 |  |  |  |
|  |  |  |  |  |  |  |
| **Total** | 855 | 51 | 825 | 49 | 1680 | 100 |

**Supplementary material 10: Number of children assessed by background characteristics of the IMCI services-providers.**

Caption: Number of children assessed by background characteristics of the IMCI services-providers by rounds of assessment.

|  | **Round 1** |  | **Round 2** |  | **All rounds** |  |
| --- | --- | --- | --- | --- | --- | --- |
|  | **n** | **%** | **n** | **%** | **n** | **%** |
| **Provider characteristics** |  |  |  |  |  |  |
| **Age** |  |  |  |  |  |  |
| **≤ 35 years** | 374 | 44 | 363 | 44 | 737 | 44 |
| **> 35 years** | 481 | 56 | 462 | 56 | 943 | 56 |
| **Missing** |  |  |  |  |  |  |
| **Sex** |  |  |  |  |  |  |
| **Male** | 388 | 45 | 385 | 47 | 773 | 46 |
| **Female** | 467 | 55 | 440 | 53 | 907 | 54 |
| **Missing** |  |  |  |  |  |  |
| **Designation** |  |  |  |  |  |  |
| **Doctor** | 199 | 23 | 198 | 24 | 397 | 24 |
| **Nurse** | 190 | 22 | 177 | 22 | 367 | 22 |
| **Paramedic** | 466 | 55 | 450 | 55 | 916 | 55 |
| **Missing** |  |  |  |  |  |  |
| **Facility characteristics** |  |  |  |  |  |  |
| **District Hospital** | 132 | 15 | 119 | 14 | 251 | 15 |
| **Sub-District Hospital** | 515 | 60 | 504 | 61 | 1019 | 61 |
| **Union-level Health Centre** | 208 | 24 | 202 | 25 | 410 | 24 |
| **Missing** |  |  |  |  |  |  |

**Supplementary material 11: Number of assessments by IMCI service-providers.**

Caption: Number of assessments by IMCI service-providers, by round.

|  | **Number of assessments** | | |
| --- | --- | --- | --- |
|  | **Round 1** | **Round 2** | **All rounds** |
| **Doctor 1** | 45 | 40 | 85 |
| **Doctor 2** | 45 | 40 | 85 |
| **Doctor 3** | 41 | 42 | 83 |
| **Doctor 4** | 35 | 42 | 77 |
| **Doctor 5** | 33 | 34 | 67 |
| **Nurse 1** | 43 | 42 | 85 |
| **Nurse 2** | 33 | 32 | 65 |
| **Nurse 3** | 44 | 34 | 78 |
| **Nurse 4** | 33 | 37 | 70 |
| **Nurse 5** | 37 | 32 | 69 |
| **Paramedic 1** | 41 | 42 | 83 |
| **Paramedic 2** | 45 | 41 | 86 |
| **Paramedic 3** | 31 | 32 | 63 |
| **Paramedic 4** | 35 | 35 | 70 |
| **Paramedic 5** | 36 | 35 | 71 |
| **Paramedic 6** | 34 | 34 | 68 |
| **Paramedic 7** | 47 | 43 | 90 |
| **Paramedic 8** | 36 | 40 | 76 |
| **Paramedic 9** | 42 | 38 | 80 |
| **Paramedic 10** | 34 | 32 | 66 |
| **Paramedic 11** | 38 | 34 | 72 |
| **Paramedic 12** | 47 | 44 | 91 |

**Supplementary material 12: Influence of several patient-, provider-, and facility-related factors on successfully conducting pulse oximetry assessments at the first attempt by IMCI services-providers**.

Caption: Influence of several patient-, provider-, and facility-related factors on successfully conducting pulse oximetry assessments at the first attempt by IMCI services-providers presented in adjusted odds ratios, N=1680.

|  |  | **Success at first attempt** | | | | | | | | |
| --- | --- | --- | --- | --- | --- | --- | --- | --- | --- | --- |
|  | **N** | **%** | **OR** | **Lower** | **Upper** | **P value** | **AOR** | **Lower** | **Upper** | **P value** |
| **Patient characteristics** |  |  |  |  |  |  |  |  |  |  |
| **Age** |  |  |  |  |  |  |  |  |  |  |
| **2-11 months** | 582 | 79.80 | Ref | Ref | Ref | Ref | Ref | Ref | Ref | Ref |
| **12-59 months** | 847 | 89.30 | 2.15 | 1.63 | 2.83 | 0.00 | 2.20 | 1.65 | 2.93 | 0.00 |
| **Sex** |  |  |  |  |  |  |  |  |  |  |
| **Female** | 634 | 82.20 | Ref | Ref | Ref | Ref | Ref | Ref | Ref | Ref |
| **Male** | 795 | 87.70 | 1.51 | 1.15 | 1.98 | 0.00 | 1.54 | 1.16 | 2.04 | 0.00 |
| **Chest indrawing** |  |  |  |  |  |  |  |  |  |  |
| **No** | 1384 | 85.60 | Ref | Ref | Ref | Ref | Ref | Ref | Ref | Ref |
| **Yes** | 38 | 79.20 | 0.59 | 0.30 | 1.19 | 0.14 | 0.68 | 0.28 | 1.65 | 0.40 |
| **Fast breathing** |  |  |  |  |  |  |  |  |  |  |
| **No** | 1250 | 85.60 | Ref | Ref | Ref | Ref | Ref | Ref | Ref | Ref |
| **Yes** | 169 | 84.90 | 0.94 | 0.62 | 1.42 | 0.77 | 1.03 | 0.30 | 3.56 | 0.96 |
| **Fever** |  |  |  |  |  |  |  |  |  |  |
| **No** | 1346 | 85.40 | Ref | Ref | Ref | Ref | Ref | Ref | Ref | Ref |
| **Yes** | 83 | 82.20 | 0.77 | 0.46 | 1.30 | 0.33 | 0.99 | 0.52 | 1.91 | 0.99 |
| **IMCI classification** |  |  |  |  |  |  |  |  |  |  |
| **No pneumonia** | 1236 | 85.60 | Ref | Ref | Ref | Ref | Ref | Ref | Ref | Ref |
| **Pneumonia/Severe pneumonia** | 186 | 84.50 | 0.91 | 0.62 | 1.35 | 0.66 | 1.06 | 0.30 | 3.78 | 0.93 |
| **Z-score (weight for height)** |  |  |  |  |  |  |  |  |  |  |
| **Not wasted (-2SD and above)** | 1287 | 85.80 | Ref | Ref | Ref | Ref | Ref | Ref | Ref | Ref |
| **Wasted (below -2SD)** | 81 | 82.70 | 0.80 | 0.46 | 1.37 | 0.41 | 0.82 | 0.47 | 1.43 | 0.48 |
| **Severely wasted (below -3SD)** | 54 | 81.80 | 0.75 | 0.40 | 1.43 | 0.39 | 0.80 | 0.41 | 1.55 | 0.50 |
| **Service provider characteristics** |  |  |  |  |  |  |  |  |  |  |
| **Age** |  |  |  |  |  |  |  |  |  |  |
| **≤ 35 years** | 625 | 84.90 | Ref | Ref | Ref | Ref | Ref | Ref | Ref | Ref |
| **> 35 years** | 804 | 85.40 | 1.05 | 0.70 | 1.57 | 0.81 | 1.08 | 0.72 | 1.62 | 0.72 |
| **Sex** |  |  |  |  |  |  |  |  |  |  |
| **Female** | 774 | 85.40 | Ref | Ref | Ref | Ref | Ref | Ref | Ref | Ref |
| **Male** | 655 | 85.00 | 0.98 | 0.65 | 1.46 | 0.90 | 0.88 | 0.54 | 1.44 | 0.61 |
| **Designation** |  |  |  |  |  |  |  |  |  |  |
| **Doctor** | 349 | 88.10 | Ref | Ref | Ref | Ref | Ref | Ref | Ref | Ref |
| **Nurse** | 302 | 82.50 | 0.64 | 0.36 | 1.12 | 0.12 | 0.60 | 0.34 | 1.05 | 0.07 |
| **Paramedic** | 778 | 85.00 | 0.77 | 0.47 | 1.26 | 0.30 | 0.82 | 0.46 | 1.45 | 0.49 |
| **Facility characteristics** |  |  |  |  |  |  |  |  |  |  |
| **District Hospital** | 221 | 88.00 | Ref | Ref | Ref | Ref | Ref | Ref | Ref | Ref |
| **Sub-District Hospital** | 863 | 84.90 | 0.77 | 0.42 | 1.41 | 0.39 | 0.91 | 0.49 | 1.72 | 0.78 |
| **Health Centre** | 345 | 84.10 | 0.73 | 0.37 | 1.42 | 0.35 | 0.76 | 0.36 | 1.62 | 0.48 |
| **Assessments** |  |  |  |  |  |  |  |  |  |  |
| **Round 1** | 727 | 85.30 | Ref | Ref | Ref | Ref | Ref | Ref | Ref | Ref |
| **Round 2** | 702 | 85.10 | 0.98 | 0.75 | 1.28 | 0.89 | 0.98 | 0.74 | 1.30 | 0.89 |

**Supplementary material 13: Influence of several patient-, provider-, and facility-related factors on successfully conducting pulse oximetry assessments within one minute by IMCI services-providers.**

Caption: Influence of several patient-, provider-, and facility-related factors on successfully conducting pulse oximetry assessments within one minute by IMCI services-providers, presented in adjusted odds ratios, N=1680.

|  |  | **Success within one minute** | | | | | | | | |
| --- | --- | --- | --- | --- | --- | --- | --- | --- | --- | --- |
|  | **N** | **%** | **OR** | **Lower** | **Upper** | **P value** | **AOR** | **Lower** | **Upper** | **P value** |
| **Patient characteristics** |  |  |  |  |  |  |  |  |  |  |
| **Age** |  |  |  |  |  |  |  |  |  |  |
| **2-11 months** | 439 | 61 | Ref | Ref | Ref | Ref | Ref | Ref | Ref | Ref |
| **12-59 months** | 709 | 75 | 1.95 | 1.58 | 2.41 | 0.00 | 2.05 | 1.65 | 2.55 | 0.00 |
| **Sex** |  |  |  |  |  |  |  |  |  |  |
| **Female** | 509 | 67 | Ref | Ref | Ref | Ref | Ref | Ref | Ref | Ref |
| **Male** | 639 | 71 | 1.20 | 0.97 | 1.48 | 0.09 | 1.21 | 0.98 | 1.50 | 0.08 |
| **IMCI classification** |  |  |  |  |  |  |  |  |  |  |
| **No pneumonia** | 998 | 70 | Ref | Ref | Ref | Ref | Ref | Ref | Ref | Ref |
| **Pneumonia/Severe pneumonia** | 144 | 66 | 0.82 | 0.61 | 1.11 | 0.20 | 0.88 | 0.32 | 2.42 | 0.81 |
| **Chest indrawing** |  |  |  |  |  |  |  |  |  |  |
| **No** | 1112 | 69 | Ref | Ref | Ref | Ref | Ref | Ref | Ref | Ref |
| **Yes** | 30 | 63 | 0.69 | 0.38 | 1.25 | 0.22 | 0.90 | 0.44 | 1.84 | 0.77 |
| **Fast breathing** |  |  |  |  |  |  |  |  |  |  |
| **No** | 1009 | 70 | Ref | Ref | Ref | Ref | Ref | Ref | Ref | Ref |
| **Yes** | 131 | 66 | 0.84 | 0.62 | 1.15 | 0.29 | 1.08 | 0.40 | 2.91 | 0.89 |
| **Fever** |  |  |  |  |  |  |  |  |  |  |
| **No** | 1083 | 69 | Ref | Ref | Ref | Ref | Ref | Ref | Ref | Ref |
| **Yes** | 65 | 65 | 0.81 | 0.53 | 1.23 | 0.32 | 0.79 | 0.49 | 1.27 | 0.33 |
| **Z-score (weight for height)** |  |  |  |  |  |  |  |  |  |  |
| **Not wasted (-2SD and above)** | 1036 | 70 | Ref | Ref | Ref | Ref | Ref | Ref | Ref | Ref |
| **Wasted (below -2SD)** | 62 | 65 | 0.80 | 0.52 | 1.23 | 0.31 | 0.79 | 0.51 | 1.24 | 0.30 |
| **Severely wasted (below -3SD)** | 44 | 67 | 0.86 | 0.51 | 1.46 | 0.58 | 0.89 | 0.52 | 1.53 | 0.67 |
| **Service provider characteristics** |  |  |  |  |  |  |  |  |  |  |
| **Age** |  |  |  |  |  |  |  |  |  |  |
| **≤ 35 years** | 488 | 67 | Ref | Ref | Ref | Ref | Ref | Ref | Ref | Ref |
| **> 35 years** | 660 | 71 | 1.18 | 0.92 | 1.51 | 0.19 | 1.17 | 0.90 | 1.53 | 0.23 |
| **Sex** |  |  |  |  |  |  |  |  |  |  |
| **Female** | 608 | 67 | Ref | Ref | Ref | Ref | Ref | Ref | Ref | Ref |
| **Male** | 540 | 71 | 1.18 | 0.92 | 1.51 | 0.20 | 1.05 | 0.78 | 1.43 | 0.74 |
| **Designation** |  |  |  |  |  |  |  |  |  |  |
| **Doctor** | 276 | 70 | Ref | Ref | Ref | Ref | Ref | Ref | Ref | Ref |
| **Nurse** | 237 | 65 | 0.78 | 0.55 | 1.11 | 0.17 | 0.77 | 0.55 | 1.08 | 0.13 |
| **Paramedic** | 635 | 70 | 0.98 | 0.72 | 1.32 | 0.88 | 1.03 | 0.73 | 1.45 | 0.86 |
| **Facility characteristics** |  |  |  |  |  |  |  |  |  |  |
| **District Hospital** | 177 | 71 | Ref | Ref | Ref | Ref | Ref | Ref | Ref | Ref |
| **Sub-District Hospital** | 701 | 70 | 0.95 | 0.65 | 1.38 | 0.78 | 1.06 | 0.72 | 1.55 | 0.78 |
| **Health Centre** | 270 | 67 | 0.83 | 0.55 | 1.26 | 0.39 | 0.81 | 0.51 | 1.29 | 0.38 |
| **Assessments** |  |  |  |  |  |  |  |  |  |  |
| **Round 1** | 560 | 67 | Ref | Ref | Ref | Ref | Ref | Ref | Ref | Ref |
| **Round 2** | 588 | 72 | 1.26 | 1.02 | 1.55 | 0.03 | 1.30 | 1.05 | 1.62 | 0.02 |

**Supplementary material 14: Influence of several patient-, provider-, and facility-related factors on adhering to SoPs while conducting pulse oximetry assessments by IMCI services-providers.**

Caption: Influence of several patient-, provider-, and facility-related factors on adhering to SoPs while conducting pulse oximetry assessments by IMCI services-providers presented in adjusted odds ratios, N=1680.

|  |  | **Adherence to SoP of PO use** | | | | | | | | |
| --- | --- | --- | --- | --- | --- | --- | --- | --- | --- | --- |
|  | **n** | **%** | **OR** | **Lower** | **Upper** | **P value** | **AOR** | **Lower** | **Upper** | **P value** |
| **Patient characteristics** |  |  |  |  |  |  |  |  |  |  |
| **Age** |  |  |  |  |  |  |  |  |  |  |
| **2-11 months** | 674 | 92.50 | Ref | Ref | Ref | Ref | Ref | Ref | Ref | Ref |
| **12-59 months** | 868 | 91.60 | 0.99 | 0.72 | 1.36 | 0.95 | 1.06 | 0.69 | 1.61 | 0.80 |
| **Sex** |  |  |  |  |  |  |  |  |  |  |
| **Female** | 719 | 93.30 | Ref | Ref | Ref | Ref | Ref | Ref | Ref | Ref |
| **Male** | 823 | 90.80 | 0.81 | 0.59 | 1.12 | 0.20 | 0.69 | 0.46 | 1.05 | 0.09 |
| **Z-score (weight for height)** |  |  |  |  |  |  |  |  |  |  |
| **Not wasted (-2SD and above)** | 1378 | 91.90 | Ref | Ref | Ref | Ref | Ref | Ref | Ref | Ref |
| **Wasted (below -2SD)** | 91 | 92.90 | 1.04 | 0.53 | 2.05 | 0.91 | 0.75 | 0.29 | 1.94 | 0.55 |
| **Severely wasted (below -3SD)** | 64 | 97.00 | 1.86 | 0.64 | 5.37 | 0.25 | 1.59 | 0.33 | 7.72 | 0.56 |
| **Chest indrawing** |  |  |  |  |  |  |  |  |  |  |
| **No** | 1488 | 92.10 | Ref | Ref | Ref | Ref | Ref | Ref | Ref | Ref |
| **Yes** | 45 | 93.80 | 0.79 | 0.33 | 1.88 | 0.59 | 0.81 | 0.19 | 3.55 | 0.79 |
| **Fast breathing** |  |  |  |  |  |  |  |  |  |  |
| **No** | 1349 | 92.40 | Ref | Ref | Ref | Ref | Ref | Ref | Ref | Ref |
| **Yes** | 179 | 89.90 | 0.66 | 0.43 | 1.03 | 0.07 | 0.60 | 0.07 | 5.43 | 0.65 |
| **Fever** |  |  |  |  |  |  |  |  |  |  |
| **No** | 1452 | 92.10 | Ref | Ref | Ref | Ref | Ref | Ref | Ref | Ref |
| **Yes** | 90 | 89.10 | 0.65 | 0.37 | 1.16 | 0.15 | 0.50 | 0.18 | 1.36 | 0.17 |
| **IMCI classification** |  |  |  |  |  |  |  |  |  |  |
| **No pneumonia** | 1334 | 92.40 | Ref | Ref | Ref | Ref | Ref | Ref | Ref | Ref |
| **Pneumonia/Severe pneumonia** | 199 | 90.50 | 0.68 | 0.44 | 1.04 | 0.08 | 1.32 | 0.14 | 12.10 | 0.81 |
| **Service provider characteristics** |  |  |  |  |  |  |  |  |  |  |
| **Age** |  |  |  |  |  |  |  |  |  |  |
| **≤ 35 years** | 686 | 93.20 | Ref | Ref | Ref | Ref | Ref | Ref | Ref | Ref |
| **> 35 years** | 856 | 91.00 | 0.74 | 0.22 | 2.49 | 0.63 | 0.29 | 0.15 | 0.53 | 0.00 |
| **Sex** |  |  |  |  |  |  |  |  |  |  |
| **Female** | 813 | 89.70 | Ref | Ref | Ref | Ref | Ref | Ref | Ref | Ref |
| **Male** | 729 | 94.60 | 2.09 | 0.64 | 6.76 | 0.22 | 2.98 | 1.34 | 6.63 | 0.01 |
| **Designation** |  |  |  |  |  |  |  |  |  |  |
| **Doctor** | 377 | 95.20 | Ref | Ref | Ref | Ref | Ref | Ref | Ref | Ref |
| **Paramedic** | 846 | 92.50 | 0.62 | 0.12 | 3.22 | 0.57 | 1.88 | 0.55 | 6.43 | 0.31 |
| **Nurse** | 319 | 87.20 | 0.36 | 0.06 | 2.04 | 0.25 | 0.42 | 0.16 | 1.10 | 0.08 |
| **Facility characteristics** |  |  |  |  |  |  |  |  |  |  |
| **District Hospital** | 243 | 96.80 | Ref | Ref | Ref | Ref | Ref | Ref | Ref | Ref |
| **Sub-District Hospital** | 943 | 92.80 | 0.39 | 0.03 | 4.79 | 0.46 | 0.03 | 0.00 | 0.25 | 0.00 |
| **Health Centre** | 356 | 86.80 | 0.21 | 0.02 | 2.67 | 0.23 | 0.00 | 0.00 | 0.05 | 0.00 |
| **Assessments** |  |  |  |  |  |  |  |  |  |  |
| **Round 1** | 725 | 85.10 | Ref | Ref | Ref | Ref | Ref | Ref | Ref | Ref |
| **Round 2** | 817 | 99.00 | 15.98 | 5.67 | 45.00 | 0.00 | 28.62 | 11.03 | 74.29 | 0.00 |

**Supplementary material 15: Influence of several patient-, provider-, and facility-related factors on agreement of identifying hypoxaemia through pulse oximetry between IMCI services-providers and study nurses.**

Caption: Influence of several patient-, provider-, and facility-related factors on agreement of identifying hypoxaemia through pulse oximetry between IMCI services-providers and study nurses, presented in adjusted odds ratios, N=1680.

|  |  | **Observed agreement (94% SpO2)** | | | | | | | | | | | | | | | |
| --- | --- | --- | --- | --- | --- | --- | --- | --- | --- | --- | --- | --- | --- | --- | --- | --- | --- |
|  | **N** | **%** | **OR** | **Lower** | | **Upper** | | **P value** | | **AOR** | | **Lower** | | **Upper** | | **P value** | |
| **Patient characteristics** |  |  |  |  | |  | |  | |  | |  | |  | |  | |
| **Age** |  |  |  |  | |  | |  | |  | |  | |  | |  | |
| **2-11 months** | 696 | 96.7 | Ref | Ref | | Ref | | Ref | | Ref | | Ref | | Ref | | Ref | |
| **12-59 months** | 905 | 97.1 | 1.23 | 0.71 | | 2.13 | | 0.47 | | 1.19 | | 0.67 | | 2.11 | | 0.55 | |
| **Sex** |  |  |  |  | |  | |  | |  | |  | |  | |  | |
| **Female** | 733 | 96.6 | Ref | Ref | | Ref | | Ref | | Ref | | Ref | | Ref | | Ref | |
| **Male** | 868 | 97.2 | 1.01 | 0.59 | | 1.75 | | 0.97 | | 1.07 | | 0.60 | | 1.89 | | 0.82 | |
| **IMCI classification** |  |  |  |  | |  | |  | |  | |  | |  | |  | |
| **No pneumonia** | 1396 | 97.5 | Ref | Ref | | Ref | | Ref | | Ref | | Ref | | Ref | | Ref | |
| **Pneumonia/Severe pneumonia** | 205 | 93.2 | 0.41 | 0.21 | | 0.80 | | 0.01 | | 0.38 | | 0.06 | | 2.40 | | 0.30 | |
| **Chest indrawing** |  |  |  |  | |  | |  | |  | |  | |  | |  | |
| **No** | 1557 | 97.1 | Ref | Ref | | Ref | | Ref | | Ref | | Ref | | Ref | | Ref | |
| **Yes** | 44 | 91.7 | 0.58 | 0.16 | | 2.12 | | 0.41 | | 1.35 | | 0.33 | | 5.53 | | 0.68 | |
| **Fast breathing** |  |  |  |  | |  | |  | |  | |  | |  | |  | |
| **No** | 1412 | 97.4 | Ref | Ref | | Ref | | Ref | | Ref | | Ref | | Ref | | Ref | |
| **Yes** | 186 | 93.5 | 0.42 | 0.21 | | 0.84 | | 0.01 | | 1.00 | | 0.16 | | 6.15 | | 1.00 | |
| **Fever** |  |  |  |  | |  | |  | |  | |  | |  | |  | |
| **No** | 1518 | 97 | Ref | Ref | | Ref | | Ref | | Ref | | Ref | | Ref | | Ref | |
| **Yes** | 83 | 95.4 | 0.78 | 0.26 | | 2.36 | | 0.67 | | 0.92 | | 0.30 | | 2.84 | | 0.89 | |
| **Z-score (weight for height)** |  |  |  |  | |  | |  | |  | |  | |  | |  | |
| **Not wasted (-2SD and above)** | 1443 | 96.9 | Ref | Ref | | Ref | | Ref | | Ref | | Ref | | Ref | | Ref | |
| **Wasted (below -2SD)** | 95 | 97.9 | 1.52 | 0.37 | | 6.15 | | 0.56 | | 1.46 | | 0.36 | | 5.95 | | 0.60 | |
| **Severely wasted (below -3SD)** | 63 | 95.5 | 0.75 | 0.22 | | 2.55 | | 0.65 | | 0.75 | | 0.21 | | 2.59 | | 0.64 | |
| **Service provider characteristics** |  |  |  |  | |  | |  | |  | |  | |  | |  | |
| **Age** |  |  |  |  | |  | |  | |  | |  | |  | |  | |
| **≤ 35 years** | 711 | 97.8 | Ref | Ref | | Ref | | Ref | | Ref | | Ref | | Ref | | Ref | |
| **> 35 years** | 890 | 96.2 | 0.67 | 0.15 | | 2.92 | | 0.60 | | 1.10 | | 0.27 | | 4.44 | | 0.89 | |
| **Sex** |  |  |  |  | |  | |  | |  | |  | |  | |  | |
| **Female** | 867 | 97.3 | Ref | Ref | | Ref | | Ref | | Ref | | Ref | | Ref | | Ref | |
| **Male** | 734 | 96.5 | 0.84 | 0.20 | | 3.49 | | 0.81 | | 0.72 | | 0.10 | | 5.31 | | 0.75 | |
| **Designation** |  |  |  |  | |  | |  | |  | |  | |  | |  | |
| **Doctor** | 384 | 98.2 | Ref | Ref | | Ref | | Ref | | Ref | | Ref | | Ref | | Ref | |
| **Nurse** | 346 | 96.6 | 0.48 | 0.05 | | 5.15 | | 0.55 | | 0.49 | | 0.06 | | 4.17 | | 0.51 | |
| **Paramedic** | 871 | 96.5 | 0.52 | 0.06 | | 4.42 | | 0.55 | | 0.30 | | 0.04 | | 2.12 | | 0.23 | |
| **Facility characteristics** |  |  |  |  | |  | |  | |  | |  | |  | |  | |
| **District Hospital** | 232 | 95.5 | Ref | Ref | | Ref | | Ref | | Ref | | Ref | | Ref | | Ref | |
| **Sub-District Hospital** | 970 | 96.5 | 1.30 | 0.23 | | 7.42 | | 0.77 | | 1.33 | | 0.15 | | 11.38 | | 0.80 | |
| **Health Centre** | 399 | 98.8 | 3.69 | 0.29 | | 47.41 | | 0.32 | | 4.97 | | 0.36 | | 68.53 | | 0.23 | |
| **Assessments** |  |  |  |  | |  | |  | |  | |  | |  | |  | |
| **Round 1** | 801 | 96 | Ref | Ref | | Ref | | Ref | | Ref | | Ref | | Ref | | Ref | |
| **Round 2** | 800 | 97.8 | 1.82 | | 1.00 | | 3.30 | | 0.05 | | 1.76 | | 0.96 | | 3.23 | | 0.07 |

**Supplementary material 16: Influence of several patient-, provider-, and facility-related factors on optimum cleanliness practices by IMCI services-providers.**

Caption: Influence of several patient-, provider-, and facility-related factors on optimum cleanliness practices by IMCI services-providers, presented in adjusted odds ratios, N=1680.

|  |  | **Adherence to cleanliness practices** | | | | | | | | |
| --- | --- | --- | --- | --- | --- | --- | --- | --- | --- | --- |
|  | **N** | **%** | **OR** | **Lower** | **Upper** | **P value** | **AOR** | **Lower** | **Upper** | **P value** |
| **Patient characteristics** |  |  |  |  |  |  |  |  |  |  |
| **Age** |  |  |  |  |  |  |  |  |  |  |
| **2-11 months** | 555 | 76.10 | Ref | Ref | Ref | Ref | Ref | Ref | Ref | Ref |
| **12-59 months** | 651 | 68.70 | 0.69 | 0.55 | 0.86 | 0.00 | 0.66 | 0.51 | 0.85 | 0.00 |
| **Sex** |  |  |  |  |  |  |  |  |  |  |
| **Female** | 566 | 73.40 | Ref | Ref | Ref | Ref | Ref | Ref | Ref | Ref |
| **Male** | 640 | 70.60 | 0.87 | 0.70 | 1.08 | 0.21 | 0.92 | 0.71 | 1.18 | 0.49 |
| **IMCI classification** |  |  |  |  |  |  |  |  |  |  |
| **No pneumonia** | 1038 | 71.90 | Ref | Ref | Ref | Ref | Ref | Ref | Ref | Ref |
| **Pneumonia/Severe pneumonia** | 160 | 72.70 | 1.04 | 0.76 | 1.43 | 0.80 | 1.17 | 0.34 | 3.98 | 0.81 |
| **Chest indrawing** |  |  |  |  |  |  |  |  |  |  |
| **No** | 1166 | 72.20 | Ref | Ref | Ref | Ref | Ref | Ref | Ref | Ref |
| **Yes** | 32 | 66.70 | 0.77 | 0.42 | 1.42 | 0.41 | 0.48 | 0.21 | 1.12 | 0.09 |
| **Fast breathing** |  |  |  |  |  |  |  |  |  |  |
| **No** | 1050 | 71.90 | Ref | Ref | Ref | Ref | Ref | Ref | Ref | Ref |
| **Yes** | 145 | 72.90 | 1.05 | 0.75 | 1.46 | 0.78 | 0.90 | 0.27 | 3.00 | 0.86 |
| **Fever** |  |  |  |  |  |  |  |  |  |  |
| **No** | 1138 | 72.20 | Ref | Ref | Ref | Ref | Ref | Ref | Ref | Ref |
| **Yes** | 68 | 67.30 | 0.79 | 0.52 | 1.22 | 0.29 | 0.59 | 0.34 | 1.02 | 0.06 |
| **Z-score (weight for height)** |  |  |  |  |  |  |  |  |  |  |
| **Not wasted (-2SD and above)** | 1074 | 71.60 | Ref | Ref | Ref | Ref | Ref | Ref | Ref | Ref |
| **Wasted (below -2SD)** | 75 | 76.50 | 1.29 | 0.80 | 2.09 | 0.29 | 1.04 | 0.59 | 1.81 | 0.90 |
| **Severely wasted (below -3SD)** | 49 | 74.20 | 1.14 | 0.65 | 2.01 | 0.64 | 1.45 | 0.74 | 2.85 | 0.28 |
| **Service provider characteristics** |  |  |  |  |  |  |  |  |  |  |
| **Age** |  |  |  |  |  |  |  |  |  |  |
| **≤ 35 years** | 569 | 77.30 | Ref | Ref | Ref | Ref | Ref | Ref | Ref | Ref |
| **> 35 years** | 637 | 67.70 | 0.61 | 0.49 | 0.77 | 0.00 | 1.07 | 0.79 | 1.46 | 0.66 |
| **Sex** |  |  |  |  |  |  |  |  |  |  |
| **Female** | 645 | 71.20 | Ref | Ref | Ref | Ref | Ref | Ref | Ref | Ref |
| **Male** | 561 | 72.80 | 1.08 | 0.87 | 1.34 | 0.48 | 0.33 | 0.23 | 0.46 | 0.00 |
| **Designation** |  |  |  |  |  |  |  |  |  |  |
| **Doctor** | 253 | 63.90 | Ref | Ref | Ref | Ref | Ref | Ref | Ref | Ref |
| **Paramedic** | 669 | 73.10 | 1.54 | 1.19 | 1.98 | 0.00 | 2.63 | 1.82 | 3.79 | 0.00 |
| **Nurse** | 284 | 77.60 | 1.96 | 1.42 | 2.70 | 0.00 | 1.77 | 1.17 | 2.69 | 0.01 |
| **Facility characteristics** |  |  |  |  |  |  |  |  |  |  |
| **District Hospital** | 91 | 36.30 | Ref | Ref | Ref | Ref | Ref | Ref | Ref | Ref |
| **Sub-District Hospital** | 823 | 81.00 | 7.50 | 5.55 | 10.13 | 0.00 | 20.34 | 13.10 | 31.60 | 0.00 |
| **Health Centre** | 292 | 71.20 | 4.35 | 3.11 | 6.08 | 0.00 | 6.96 | 4.14 | 11.73 | 0.00 |
| **Assessments** |  |  |  |  |  |  |  |  |  |  |
| **Round 1** | 512 | 60.10 | Ref | Ref | Ref | Ref | Ref | Ref | Ref | Ref |
| **Round 2** | 694 | 84.10 | 3.52 | 2.79 | 4.44 | 0.00 | 4.82 | 3.68 | 6.32 | 0.00 |

**Supplementary material 17: Summary of findings on the secondary research questions.**

Caption: Summary of findings on the secondary research questions based on paper objectives.

| **Outcome** |  | **Secondary Research Questions** | **Answers** |
| --- | --- | --- | --- |
| Feasibility | 1 | Can they successfully conduct pulse oximetry assessments in two attempts? | 96%, 95% CI (95,97) |
|  | 2 | Can they successfully conduct pulse oximetry assessments in three attempts? | 99%, 95% CI (98,99) |
|  | 3 | Do various patient-, provider- and facility- factors influence successfully conducting pulse oximetry assessments at the first attempt? | Supplementary material 13 |
|  | 4 | Is there any variation in successfully conducting pulse oximetry assessments at the first attempt by individual IMCI service-providers? | Figure-3 |
|  | 5 | Can IMCI service-providers successfully conduct pulse oximetry assessments in three minutes? | 94%, 95% CI (92,95) |
|  | 6 | Can MCI service-providers successfully conduct pulse oximetry assessments in five minutes? | 98%, 95% CI (97,98) |
|  | 7 | Do various patient-, provider-, and facility- factors influence successfully conducting pulse oximetry assessments within one minute? | Supplementary material 14 |
|  | 8 | Is there any variation in successfully conducting pulse oximetry assessments within one minute by individual IMCI-service providers? | Figure-3 |
|  | 9 | What is the performance time for successfully conducting pulse oximetry assessments? | Figure-4 |
|  | 10 | Do various patient-, provider-, and facility-related factors influence performance time for successfully performing pulse oximetry | Figure-4 |
|  | 11 | Is there any variation in performance time for successfully performing pulse oximetry by individual IMCI service-providers? | Figure-4 |
|  |  |  |  |
| Fidelity | 12 | Do the IMCI service providers ensure the baby was calm before conducting pulse oximetry assessment? | 93%, 95% CI (91,94) |
|  | 13 | Do the IMCI service providers place the probe appropriately while conducting pulse oximetry assessments? | 99%, 95% CI (98,99) |
|  | 14 | Do various patient-, provider-, and facility- factors influence adhering to Standard Operating Procedure (SoP) while conducting pulse oximetry assessments? | Supplementary material 15 |
|  | 15 | Is there any variation in adhering to Standard Operating Procedure (SoP) while conducting pulse oximetry assessments by individual IMCI service-providers? | Figure-3 |
|  | 16 | Do the IMCI service-providers adhere to cleanliness practices before conducting pulse oximetry assessments? | 72%, 95% CI (70,74) |
|  | 17 | Do various patient-, provider-, and facility- factors influence adhering to cleanliness practices before conducting pulse oximetry assessments? | Supplementary material 17 |
|  | 18 | Is there any variation in adhering to cleanliness practices before conducting pulse oximetry assessment by individual IMCI service-providers? | Supplementary material 19 |
|  | 19 | Do various patient-, provider-, and facility-related factors influence agreement of hypoxaemia identification (SpO2<94%) through pulse oximetry? | Supplementary material 16 |
|  | 20 | Is there any variation in agreement of hypoxaemia identification (SpO2<94%) through pulse oximetry by individual IMCI service-providers? | Figure-3 |
